# Two Case Reports of a Malignant Germ Cell Tumor of Ovary and a Granulosa Cell Tumor: Interest of Tumoral Immunochemistry in the Identification and Management

**DOI:** 10.3389/fonc.2014.00097

**Published:** 2014-05-09

**Authors:** J. Bouquet de Jolinière, N. Ben Ali, A. Fadhlaoui, J. B. Dubuisson, L. Guillou, A. Sutter, D. Betticher, H. M. Hoogewoud, A. Feki

**Affiliations:** ^1^Department of Obstetrics and Gynecology, Hôpital Fribourgeois, Fribourg, Switzerland; ^2^Argot Laboratory Lausanne, Department of Pathology and Cytology, Lausanne, Switzerland; ^3^Department of Oncology, Hôpital Fribourgeois, Fribourg, Switzerland; ^4^Department of Radiology, Hôpital Fribourgeois, Fribourg, Switzerland

**Keywords:** germ cell tumors, dysgerminoma, teratoma, yolk sac, ovarian cell tumor, immunohistochemistry

## Abstract

**Objective:** In this article, we present two case reports. The first case was a malignant germ cell tumor of the right ovary in a 23-year old woman and the second case was a bilateral undifferentiated granulosa cell tumor in a 71-year old woman. The aim of these reports is to illustrate the interest of the immunohistochemical analysis to define the correct diagnosis, to better classify these ovarian tumors and improve their management.

**Methods:** In this study, we report two cases. The first case concerns a 23-year old woman (A) with a mixed germ cell tumor of the right ovary [dysgerminoma (75%), yolk sac tumor (20%), and a mature teratoma (5%)], and the second case concerns a 71-year old woman (B) with a bilateral non-differentiated and necrotic granulosa cell tumor of both ovaries. The staging system was used according to both the classifications: International Federation of Gynaecology and Obstetrics 1987 for ovarian cancer and TNM code 2009.

**Results:** The immunostaining establishes the malignancy and the immunochemistry contributes to confirm effectively the right diagnosis (Tables 2 and 3).

**Conclusion:** An immunohistochemical analysis is mandatory for the choice of chemotherapy to obtain a better response of the disease and improve the survival prognosis. The efficiency of the chemotherapy authorizes a conservative surgery including a unilateral salpingo-oophorectomy preserving fertility (A). Concerning the non-dysgerminoma tumor (B), and after a surgical staging and debulking, chemotherapy was recommended. The type of tumor and its histological feature conditioned the choice of treatment. The benefit of the immunohistological analysis in this case allowed the right adjuvant treatment.

## Introduction

Malignant ovarian germ cell tumors (OGCTs) comprise only 2–5% of all ovarian cancers. They are different significantly from epithelial ovarian cancers. They are concerned with women at the childbearing age and a better prognosis when compared with epithelial counterparts. Moreover, these patients will retain their fertility after multimodal treatment. It is the reason why an accurate diagnosis, staging, and treatment must be realized early to improve the prognosis, the results, and their fertility future.

The treatment outcomes also depend on the stage of disease at the moment of diagnosis. Chemosensitivity is also dependent on the types of OGCT and differing prognoses.

### Epidemiology

Ovarian germ cell tumors are very rare ovarian tumors, up to 5% of malignancies and exceptional over the age of 40 years. Before the menarche and between 13 and 20 years, 90% of ovarian tumors are OGCTs, 60% of them are of germ cell origin, with 65% being malignant (1). It is very difficult to define associated etiological factors (2). But according to Teter and Boczkowski (3), it seems that the presence of dysgenic ovaries is a predisposing factor to dysgerminoma development. Mandel et al. described familial cases (4). A Norwegian prospective study in 1997 showed some evidence that pregnancy is sometimes associated with an increased risk of developing an OGCT (5).

### Pathology

In the embryo, germ cells appear in the wall of the yolk sac, and then migrate to the genital ridge. They incorporate into the developing gonad. OGCT are derived from the differentiation of these germ cells into somatic tissues (teratoma), germinal epithelium (dysgerminoma), extra-embryonic trophoblast (choriocarcinoma), and yolk sac (yolk sac tumor) (2).

According to WHO, germ cell tumors are classified as dysgerminomas or non-dysgerminomas. Dysgerminomas are the most common (50%) and considered to be the female equivalent of testicular seminomas. However, mixed component germ cell tumors accounted for 10% of all germ tumors.

In case of non-dysgerminomas, these tumors are known to form embryonic or extra-embryonic histological patterns. Yolk sac tumors and embryonic carcinoma (endodermal sinus tumors) are highly malignant and represent 20% of OGCTs (2). The bilateralism of these tumors is rare, and they grow rapidly in the intra-peritoneal space secreting AFP. It is usual that non-dysgerminomas were found to be associated with immature teratoma that contains components derived from all three primitive germ layers. The identification of more than one element in the tumor implies treatment modification (2). Lastly, other tumors like embryonic carcinoma, choriocarcinoma, and polyembryoma are classified as non-dysgerminomas.

The benefit of immunohistochemistry (IHC) was shown to be evidence-based. All these tumors were known to produce serum markers that are useful for pre-operative workup, diagnosis, and monitoring of both disease progression and response to treatment (6). AFP and β-hCG are produced in some of the germ cell tumors. Dysgerminomas produce commonly lactate dehydrogenase (LDH) and also sometimes HCG. When present, this marker suggests a mixed germ cell tumor.

## Materials and Methods

This study includes a report of two cases.

### Presenting signs and symptoms

The first case concerned a 23-year old young woman (A) with a mixed germ cell tumor of right ovary. The tumor was composed of 75% dysgerminoma, 20% yolk sac tumor, and 5% mature teratoma. This patient was virgo and desires a future pregnancy with an impact on her management. She was admitted in an emergency with the most frequent primary symptoms: abdominal pain, an enormous palpable pelvic mass on examination, and an abdominal distension. The duration of symptoms ranged from 3 months.

The second case (B) was a 71-year old woman with a bilateral non-differentiated and necrotic granulosa cell tumor. A previous total hysterectomy with bilateral adnexectomy was performed a few years ago for a benign tumor. She has presented the same symptoms associated with 1-year history of abdominal distension for the second nausea and vomiting, urinary symptoms with bilateral kidney dilatation, and hypertension. The co morbidities were obesity, cardiac disease (left ventricular insufficiency), kidney insufficiency, and bilateral pleural effusion. These symptoms occurred since 11 years.

In the two cases, the staging system was used according to the classification of International Federation of Gynaecology and Obstetrics (FIGO) 1987 for ovarian cancer and TNM code 2009. The clinical picture depends on the rapidity of tumor growth. If dysgerminomas are growing slower, they may present with non-specific abdominal symptoms (2).

## Results

### Diagnostic imaging

Abdominal ultrasound was the first and most common investigation performed in these two patients.

The first patient (A) presented a bilateral kidney dilatation, probably due to a mechanical and external compressive process. The size of right ovary was 16 cm in diameter with a normal left ovary and without ascites.

An abdominal magnetic resonance imaging was performed showing a pelvic mass of 16 cm diameter, an important thickness (solid aspect), with a second central tumor inside with a hemorrhagic portion.

Concerning the second patient (B), ultrasounds and CT scan showed a very voluminous intra abdominal mass with ascites, peritoneal and omentum metastasis nodules, and a bilateral kidney dilatation during the urinary time. Ultrasound imaging showed an irregular thickness on the bladder wall, a heterogeneous and flaking aspect of the abdominal mass with hyper vascular signs on Doppler.

### Tumor markers

In the pre-operative workup, the dosages of serum markers AFP, HCG, AMH, beta HCG, and LDH are useful because the germ cell tumors are produced by them. These tumor markers have a role in diagnosis, monitoring progression, and response to treatment.

Dysgerminomas produce LDH and sometimes HCG. In this case, HCG suggests the presence of a mixed germ cell that the tumor contains. A choriocarcinoma can produce HCG and endodermal sinus tumors AFP.

Finally, depending on their composition, the germ cell tumor can be associated with elevation of either or both tumor markers (6–8).

The first patient (A) had AMH (7–9 pmol/l), LDH elevated (3861 UI/l), beta HCG (118 UI/l), CA-125 (136 UI/ml), E2 (310 pmol/l), and AFP (50 ng/ml). The tumor corresponded to a germ cell tumor (dysgerminoma 75%, yolk sac tumor 25%, and teratoma 5%).

The second patient (B) had CA-125 (266 UI/ml), CA-19.9 (7 UI/ml), CEA (6.2 ng/ml), CA-15.3 (18.4 UI/ml), LDH (1.17 UI/ml), and HCG (2.5 UI/ml).

### Surgical management

Surgery was the first treatment for these two patients. A fertility-sparing operation was defined for the patient (A) as the preservation of the uterus and one ovary in order to maintain fertility (9). Radical surgery included appendectomy for the patient (B).

The principles of cytoreductive surgery in ovarian cancer must be applied to the management of OGCTs. But, for a young girl desiring a pregnancy, the surgical treatment must be conservative, without danger for the survival prognosis and preserve fertility.

In a series of 22 patients, Weinberg et al. (10) described 8 with a spontaneous pregnancy for a fertility rate of 80%, resulting in 11 live full-term births.

The potential after fertility-sparing surgery and post-operative chemotherapy is excellent.

There is a general consensus for a midline incision, unilateral salpingo-oophorectomy, and a complete staging of the peritoneal cavity, with an appendicectomy and omentectomy.

At the beginning of the surgical process, cytology must be performed, with biopsies of any suspicious areas in the peritoneum. A bulky residual disease reduces the effect of chemotherapy and the survival (11). At last, the largest study addressing the issue of routine lymph node sampling did not produce clear evidence that this was advantageous (12).

Bailey and Church (2) state that the extent of surgical resection requires careful judgment and knows that the debulking procedure must be achieved with acceptable surgical morbidity allowing the excellent impact of chemotherapy in OGCTs.

- The young patient (A) was operated by an esthetic Pfannenstiel incision, and a midline incision under the skin to obtain a large space for removing the mass without rupture.

The technique consists to exteriorize the tumor outside from the peritoneal cavity, allowing the right salpingo-oophorectomy, the ligature of vascular pedicles. The pathologist is in the operating theater. Cytology was performed at first.

**Figure 1:**
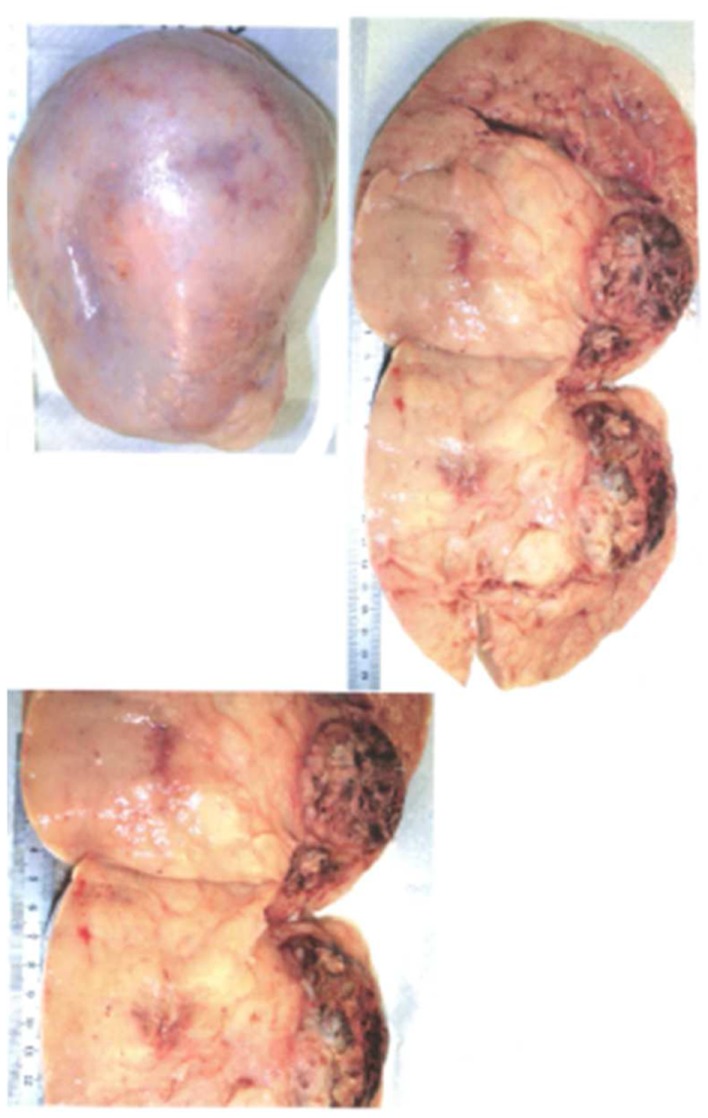
**Macroscopic view of germ cell tumor**.

The tumor weight was 3700 g, 16 cm × 13 cm diameter, 11 cm of thickness. The capsule is smooth and shining, white color with a cerebral aspect, without rupture visible macroscopically (Figure 1). After cutting into two parts, a yellow tumor appeared with a hemorrhagic and necrotic center nearing the ovarian hilum (Figure 1).

Many peritoneal biopsies were performed beside right and left bowels, inside the cul-de-sac of Douglas, under the diaphragm and partial omentectomy. The left ovary was normal.

- The second patient (B) 71 years old presented on emergencies an enormous abdominal mass with a sub occlusion and many co morbidities: a chronic ischemic heart disease (FEVG 30%), acute renal failure multifactorial with a bilateral ureteral compression by the pelvic mass, iron deficiency anemia, and a respiratory failure with pleural effusion.

The pose by cystoscopy and UPR of pre-operative probes (double J) has facilitated ureteral diuresis (no suspicious lesions endo vesical).

A midline above and below the umbilicus allowed access to the peritoneal cavity and the discovery of a voluminous tumor (>13 cm diameter), intra-peritoneal bleedings (300 cc), and bowel adhesions. The tumor compressed the rectum and the bowel sigmoid.

Seen that the patient have had a previous surgery (total hysterectomy and bilateral adnexectomy), the origin seemed to be from the retro peritoneum or ovarian remnants.

A metastasis is discovered on the root of mesentery, 3 cm diameter at the departure of inferior mesentery artery. A biopsy is realized.

**Figure 2:**
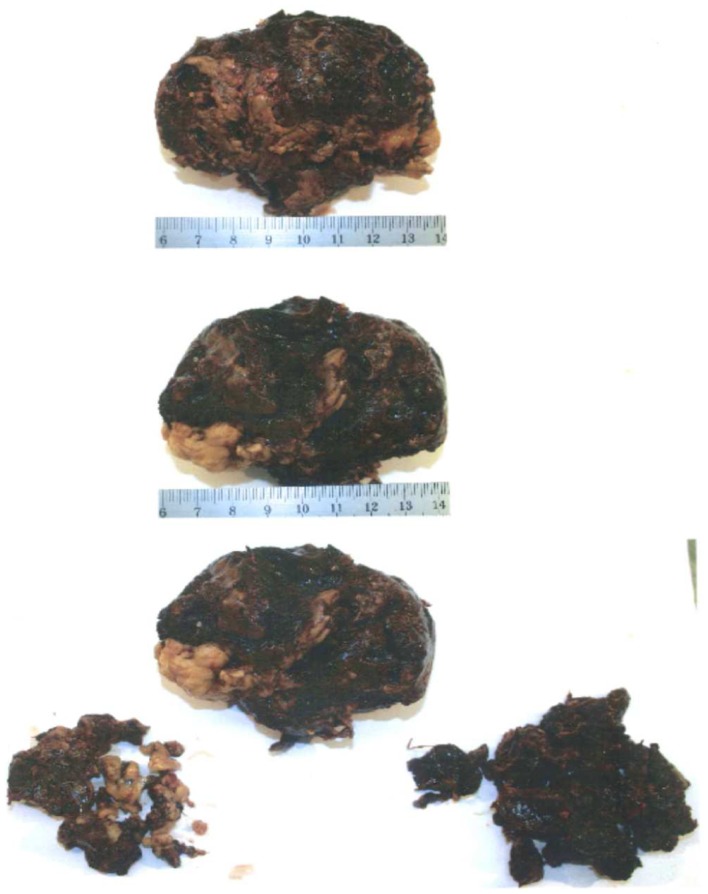
**Macroscopic view of granulosa tumor**.

The tumor (Figure 2) is removed with an appendectomy but the bleedings obliged to let inside a packing to perform hemostasis. An embolization was realized at the same time.

A reoperation was performed 24 h later to remove the packing.

### Histology

The interest of these reports is to understand the difficulties to have an optimal diagnosis to perform the management.

The risk of malignancies is between 20 and 55% reported in previous studies (13).

If the diagnosis is not possible macroscopically, the microscopic examination is always useful. Helped by reasoning on immunochemistry identification, diagnosis is obtained by elimination.

- The case (A) is concerning a 16 cm × 13 cm long left ovary (Figure 1). The intraoperative frozen section showed a dysgerminoma. The immunochemistry helped to refine the diagnosis (Table 1), and confirmed the germ cell tumor with the percentage of dysgerminoma, yolk sac tumor, and mature teratoma.
- For the patient (B), the first cytological and morphologic aspect evoked a tumor of sex cord stromal ovarian origin. But the arrangement vascular evoked also a neuro endocrinal tumor (PNET: neuro ectodermal primitive tumor) and the morphologic aspect a synovial sarcoma.

**Table 1:** **Markers of germ cell tumor (A)**.

Markers	Dysgerminoma seminoma 75%	Yolk sac tumor 25%	Mature teratoma 5%
Cytokeratines AE1/AE3	−	+	
PLAP	+	−	
CD 117	+	25% +	
CD 30	10% +	−	
EMA	30% +	5% +	
CA-19.9	10% +	20% +	
Vimentin (stroma)	100% +	100% +	
CA-125	20% +	10% +	
CD 10	−	−	
CK 7	−	20% +	

The others possible diagnosis could have been an endometrial sarcoma, and with the histo morphological aspect a malignant adrenal tumor, finally a small cell carcinoma hypercalcemic type.

In this case, the immunostaining (NSE) confirms the malignancy and the immunochemistry allows the reasoning to determine effectively the right diagnosis (Tables 2 and 3).

The final diagnosis is a granulosa tumor of ovary with 40% of necrosis.

### Immunochemistry

Immunohistochemistry is a wide-used biological technique that combines anatomy, physiology, immunology, and biochemistry (14–16).

The procedure is using the antigen–antibody binding reaction and in this case the IHC can be considered as a method that visualizes distribution and localization of specific antigen or cellular components in separated tissues or tissue sections (14). Compared to other bio-techniques using antigen–antibody reactions such as immuno-precipitation, or western-blot, IHC provides *in situ* information, which promises a more efficient experimental result.

These techniques require at least three sequences:

(1) a primary antibody, which is binding to specific antigen;
(2) the antibody–antigen complex formed by incubation with a secondary enzyme-conjugated antibody;
(3) then, the enzyme in the presence of substrate and chromogen catalyzes to generate colored deposits at the sites of antibody–antigen binding.

The results are detailed for each case in Table 1 for the case (A) and Tables 2 and 3 for the case (B).

**Table 2:** **Negative markers of tumor (B)**.

Synaptophysine chromogranine	−	Eliminate endocrine tumors
Cytokeratines 20/7	−	
TTF1	−	Eliminate lung tumors
Melan-A HMB 45	−	Eliminate adrenocortical tumors
Cytokeratines AE1/AE3	−	
CD45 LCA	−	Eliminate lymphomas
Cytokeratines 8/9 CD 10	−	
CA-125	−	
WT1	−	

**Table 3:** **Positive markers of tumor (B)**.

Calretinin vimentin inhibin CD 99	80% +	In according with a granulosa tumor of ovary
SMA BCL2	50% +	In according with a granulosa tumor of ovary

## Discussion

In view of the high risk of malignancy, ovarian masses should be managed as though they were malignant until proven otherwise. But, often, the diagnosis of malignancy is not obvious, following the tumor nature.

The results about these two cases are demonstrative and illustrate the difficulties to obtain the correct diagnosis. The following management and consequently the prognosis depend on it. The prognosis of OGCT has improved vastly over the last 30 years due to modern chemotherapy (2).

The reason why a correct immunohistochemical examination is necessary explains the choice of chemotherapy used to obtain the best response and improve the prognosis of survival. The efficacy of chemotherapy now allows conservative surgery including only a unilateral salpingo-oophorectomy preserving fertility (A).

Krishnansu Tewari reports that the evolution of systemic treatment for ovarian germ cell cancer (A) has paralleled the advances made in the treatment of testicular germ cell cancers because of the biologic similarity between ovarian and testicular germ cell tumors (17, 18). Furthermore, all data confirmed that with an effective adjuvant therapy (chemotherapy) (19) and a conservative surgery, most women with ovarian germ cell malignancies have a good survival rate of 93%. They will be able to retain their menstrual and reproductive potential after treatment (17).

Concerning other non-dysgerminoma tumors (B), after a surgical staging and treatment, chemotherapy is delivered in front-line (BEP: cysplatin, etoposide, bleomycin). The choice of treatment is conditioned by the type of tumor and its histological feature.

In case of this patient (B), the immuno-histological reasoning allowed the right adjuvant post-operative treatment (10).

## Conflict of Interest Statement

The authors declare that the research was conducted in the absence of any commercial or financial relationships that could be construed as a potential conflict of interest.

## Supplementary Material

The Supplementary Material for this article can be found online at http://www.frontiersin.org/Journal/10.3389/fonc.2014.00097/abstract

## References

[1] Barber HRK Managing ovarian tumors of childhood and adolescence. 3rd ed Ovarian Carcinoma-Aetiology, Diagnosis and Treatment. New York, NY: Springer-Verlag (1993).

[2] Bailey J Church D Management of germ cell tumors of the ovary. Rev Gynaecol Pract (2005) 5:201–6 10.1016/j.rigp.2005.09.001

[3] Teter J Boczkowski K Occurrence of tumors in dysgenetic gonads. Cancer (1967) 20:1301–10 10.1002/1097-0142(196708)20:8<1301::AID-CNCR2820200814>3.0.CO;2-4 4291636

[4] Mandel M Toren A Kende G Neuman Y Kenet G Rechavi G Familial clustering of malignant germ cell tumors and Langerhans histiocytosis. Cancer (1994) 73:1980–3 10.1002/1097-0142(19940401)73:7<1980::AID-CNCR2820730732>3.0.CO;2-9 8137225

[5] Albrektsen G Heuch I Kvale G Full term pregnancy and incidence of ovarian cancer of stromal and germ cell origin: a Norwegian prospective study. Br J Cancer (1997) 75:767–70 10.1038/bjc.1997.136 9043039 PMC2063347

[6] Dark GG Bower M Newlands ES Paradinas F Rustin GJ Surveillance policy for stage 1 ovarian germ cell tumors. J Clin Oncol (1997) 15(2):620–4 9053485 10.1200/JCO.1997.15.2.620

[7] Schultz KA Sencer SF Messinger Y Neglia JP Steiner ME Pediatric ovarian tumors: a review of 67 cases. Pediatr Blood Cancer (2005) 44:167–73 10.1002/pbc.20233 15490488

[8] De Backer A Madern GC Oosterhuis JW Hakvoort-Cammel FG Hazebroek FW Ovarian germ cell tumors in children: a clinical study of 66 patients. Pediatr Blood Cancer (2006) 46:459–64 10.1002/pbc.20633 16206211

[9] Tangir J Ovarian germ cell malignancies. Obstet Gynecol (2003) 101:2 10.1016/s0029-7844(02)02508-5 12576247

[10] Weinberg LE Lurain JR Sing DK Schink JC Survival and reproductive outcomes in women treated for malignant ovarian germ cell tumors. Gynecol Oncol (2011) 121:285–9 10.1016/j.ygyno.2011.01.003 21256579

[11] Slayton RE Park RC Silverberg SG Shingleton H Creasman WT Blessing JA Vincristine, dactinomycin, and cyclophosphamide in the treatment of malignant germ cell tumors of the ovary. A Gynecologic Oncology Group Study (a final report). Cancer (1985) 56:243–8 10.1002/1097-0142(19850715)56:2<243::AID-CNCR2820560206>3.0.CO;2-T 2988740

[12] Williams S Blessing JA Liao SY Ball H Hanjani P Adjuvant therapy of ovarian germ cell tumors with cisplatin, etoposide, and bleomycin: a trial of the Gynecologic Oncology Group. J Clin Oncol (1994) 12:701–6 7512129 10.1200/JCO.1994.12.4.701

[13] Smith HO Berwick M Verschraegen CF Wiggins C Lansing L Muller CY Incidence and survival rates for female malignant germ cell tumors. Obstet Gynecol (2006) 107:1075–85 10.1097/01.AOG.0000216004.22588.ce 16648414

[14] Dimopoulos MA Papadopoulou M Andreopoulou E Papadimitriou C Pavlidis N Aravantinos G Favorable outcome of ovarian germ cell malignancies treated with cisplatin or carboplatin-based chemotherapy: a Hellenic Cooperative Oncology Group study. Gynecol Oncol (1998) 70:70–4 10.1006/gyno.1998.5047 9698477

[15] Billmire D Vinocur C Rescorla F Outcome and staging in malignant germ cell tumors of the ovary in children and adolescents: an intergroup study. J Pediatr Surg (2004) 39:424–9 10.1016/j.jpedsurg.2003.11.027 15017564

[16] Sino Biological Inc. Available from: http://www.protocolonline.org/prot/Immunology/Immunohistochemistry/

[17] Gershenson DM Management of early ovarian cancer: germ cell and sex cord-stromal tumors. Gynecol Oncol (1994) 55:S62–72 10.1006/gyno.1994.1343 7530680

[18] Tewari K Cappuccino F Disaia PJ Berman ML Manetta A Kohler MF Treatment of germ cell tumors. Obstet Gynecol (2000) 95:1 10.1016/S0029-7844(99)00470-6 10636515

[19] Talerman A Germ cell tumors of the ovary. 4th ed In: Kurman RJ, editor. Blaustein’s Pathology of the Female Genital Tract. New York, NY: Springer-Verlag (1994). p. 849–914

