# Supplementary figures and images for: Two Case Reports of a Malignant Germ Cell Tumor of Ovary and a Granulosa Cell Tumor: Interest of Tumoral Immunochemistry in the Identification and Management
Source: Front Oncol. 2014 May 9;4:97. doi: 10.3389/fonc.2014.00097 (PMC4055855; doi:10.3389/fonc.2014.00097)

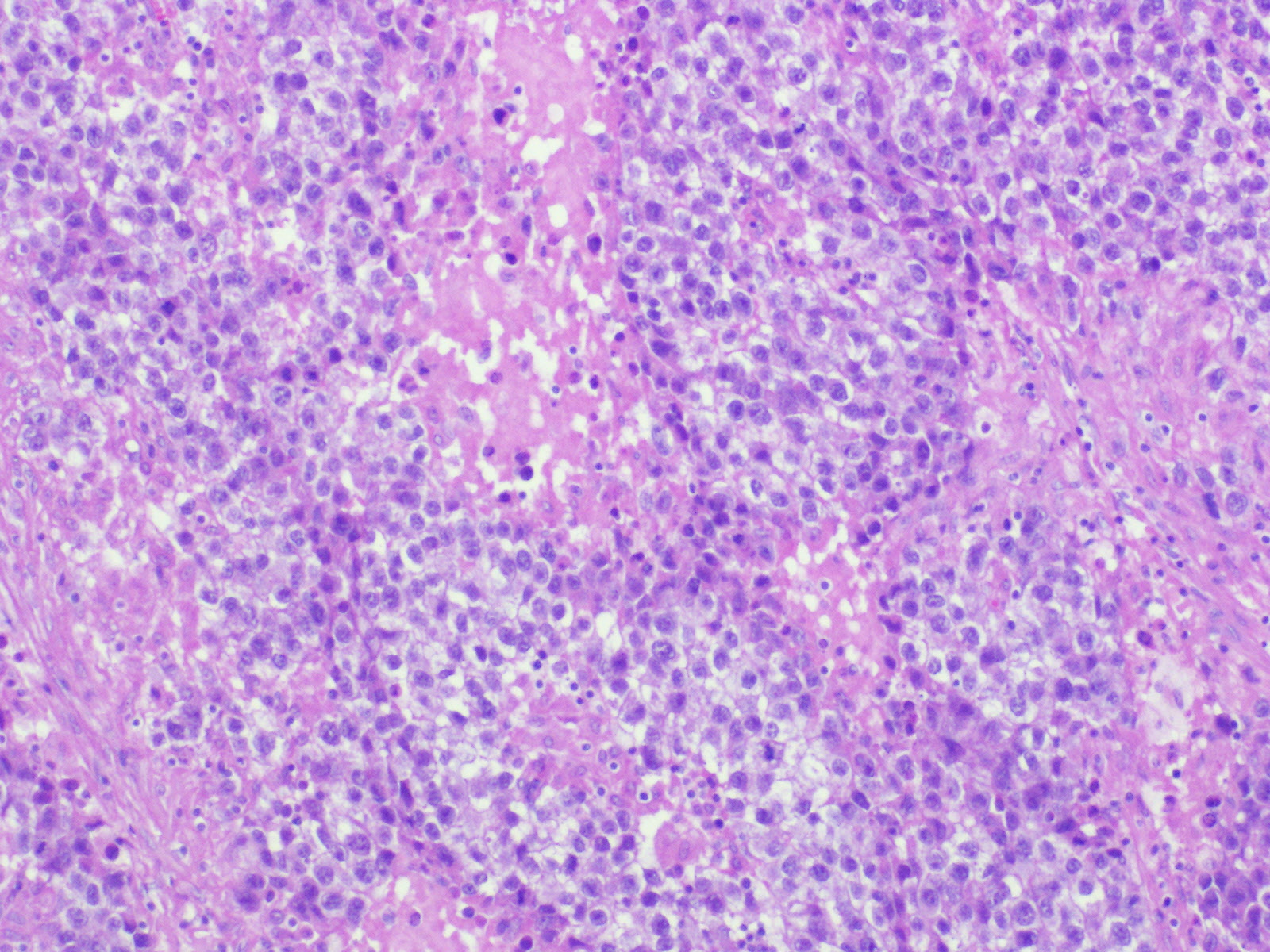

Supplement: Supplementary file 1 [file Presentation1.ZIP › 1 Composante dysgerminome.JPG]

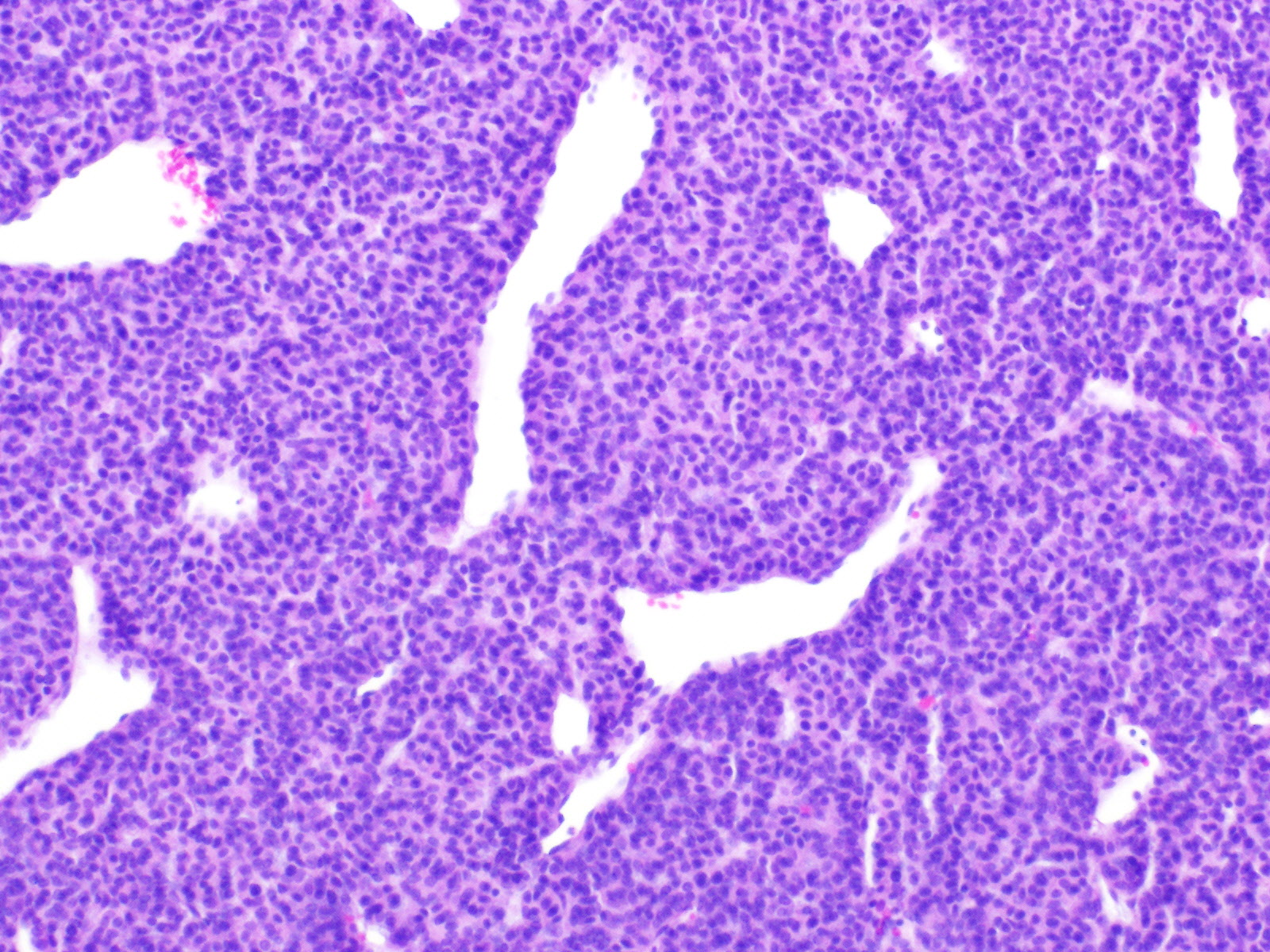

Supplement: Supplementary file 1 [file Presentation1.ZIP › 1 tum granulosa HE x 10.JPG]

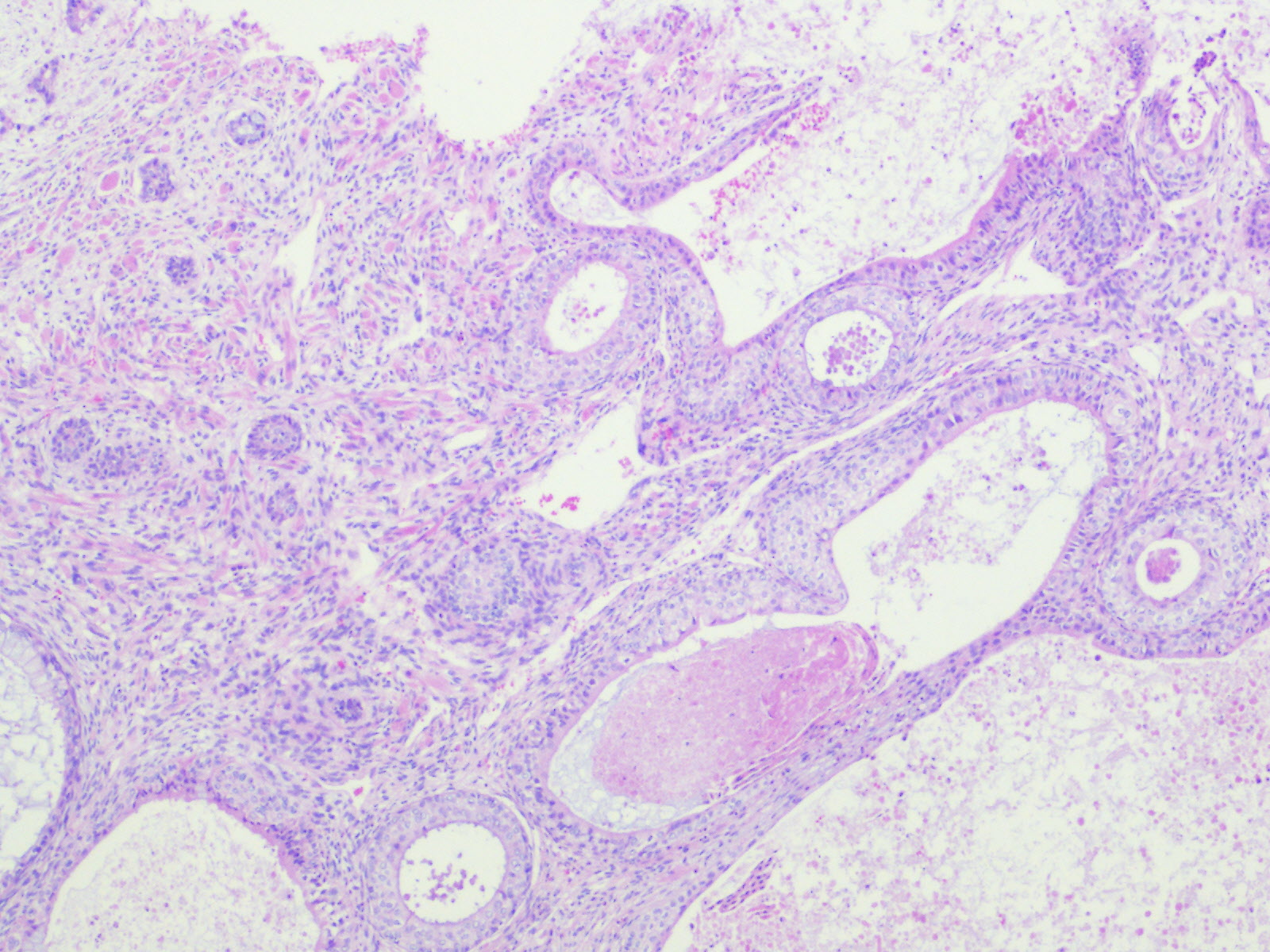

Supplement: Supplementary file 1 [file Presentation1.ZIP › 10 composante tératome immature.JPG]

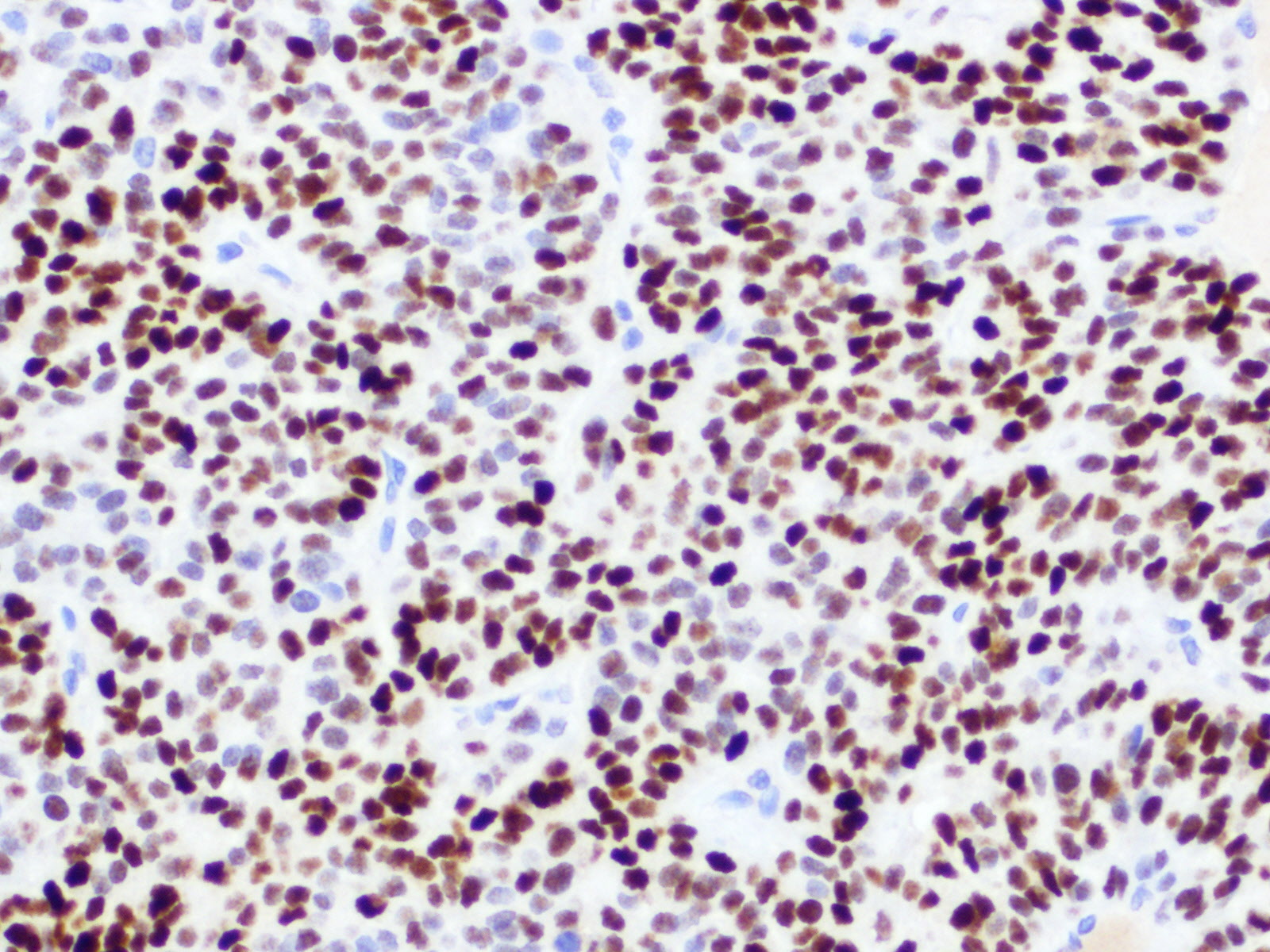

Supplement: Supplementary file 1 [file Presentation1.ZIP › 10 Tum granulosa PR pos.JPG]

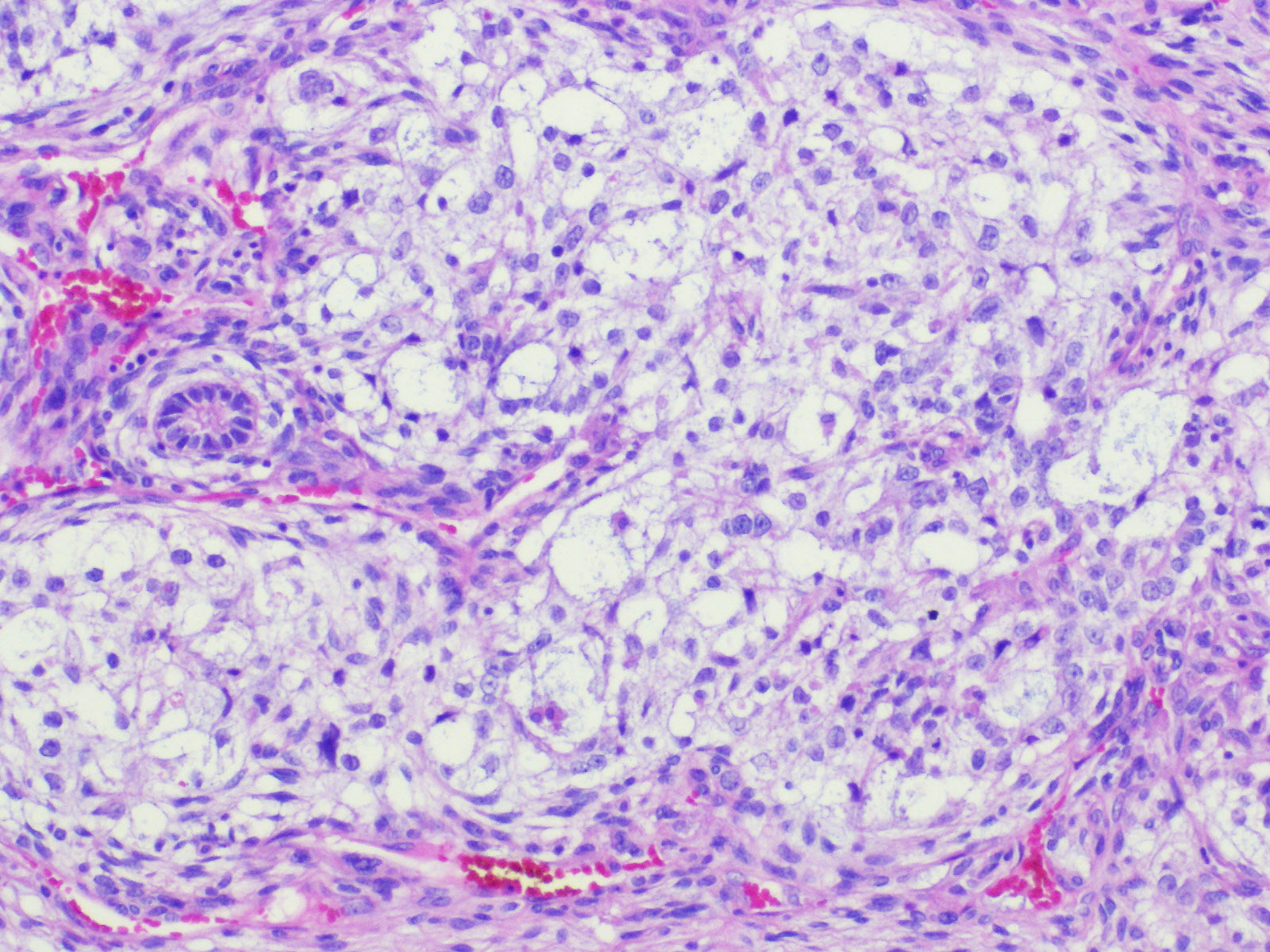

Supplement: Supplementary file 1 [file Presentation1.ZIP › 11 composante tum vittelline.JPG]

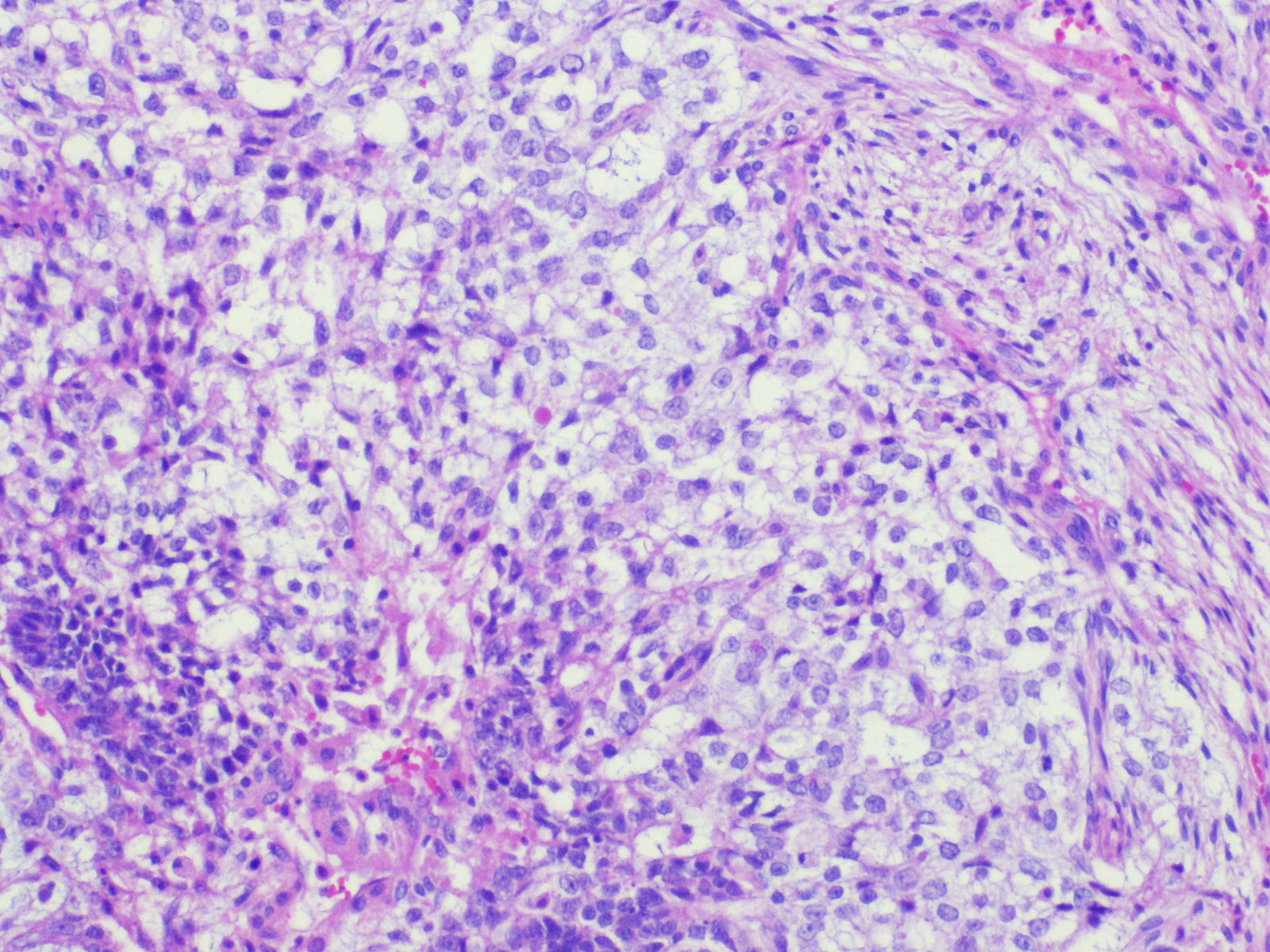

Supplement: Supplementary file 1 [file Presentation1.ZIP › 12 composante tum vitelline.JPG]

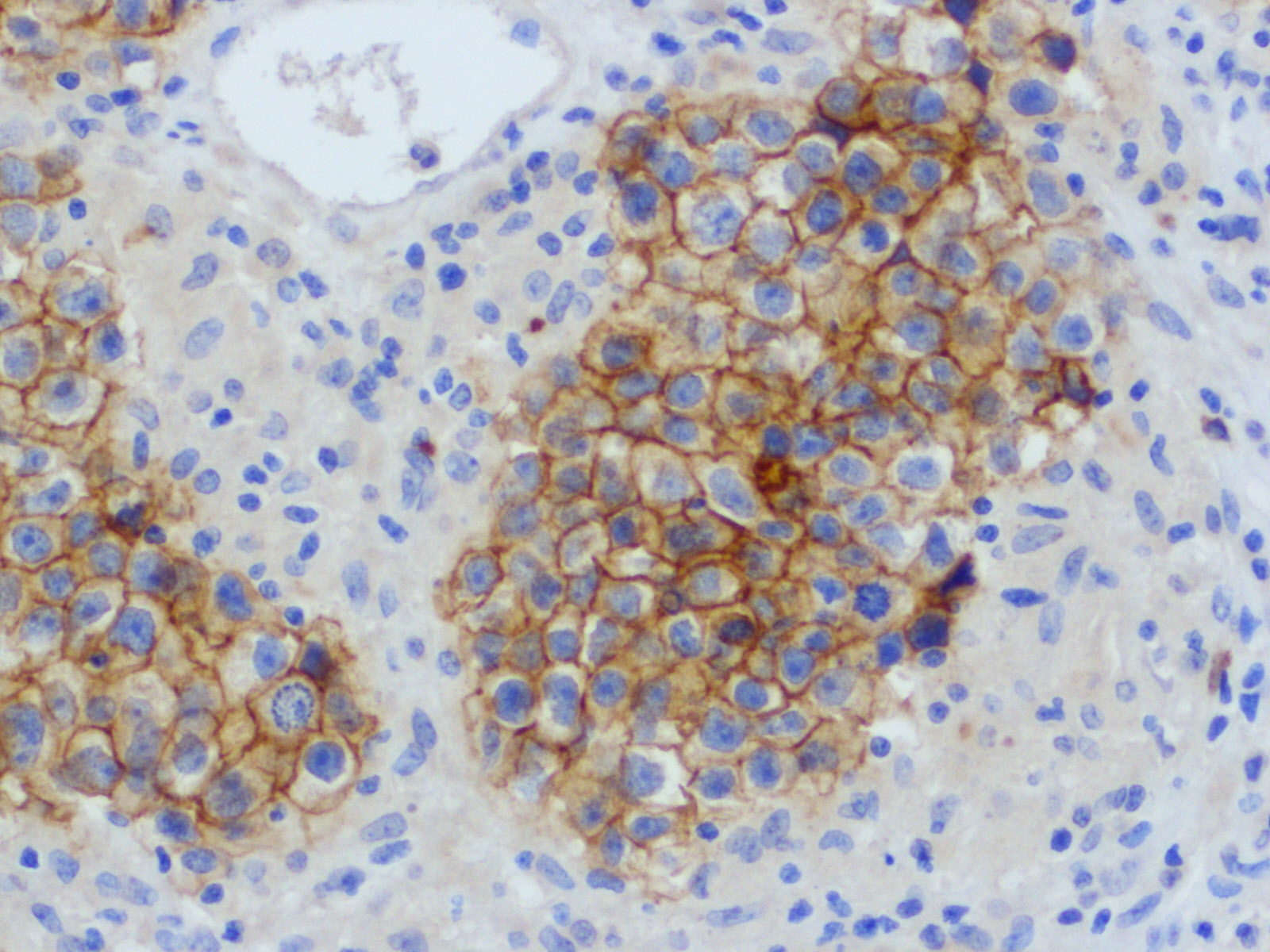

Supplement: Supplementary file 1 [file Presentation1.ZIP › 13 composante dysgerminome, CD117 pos.JPG]

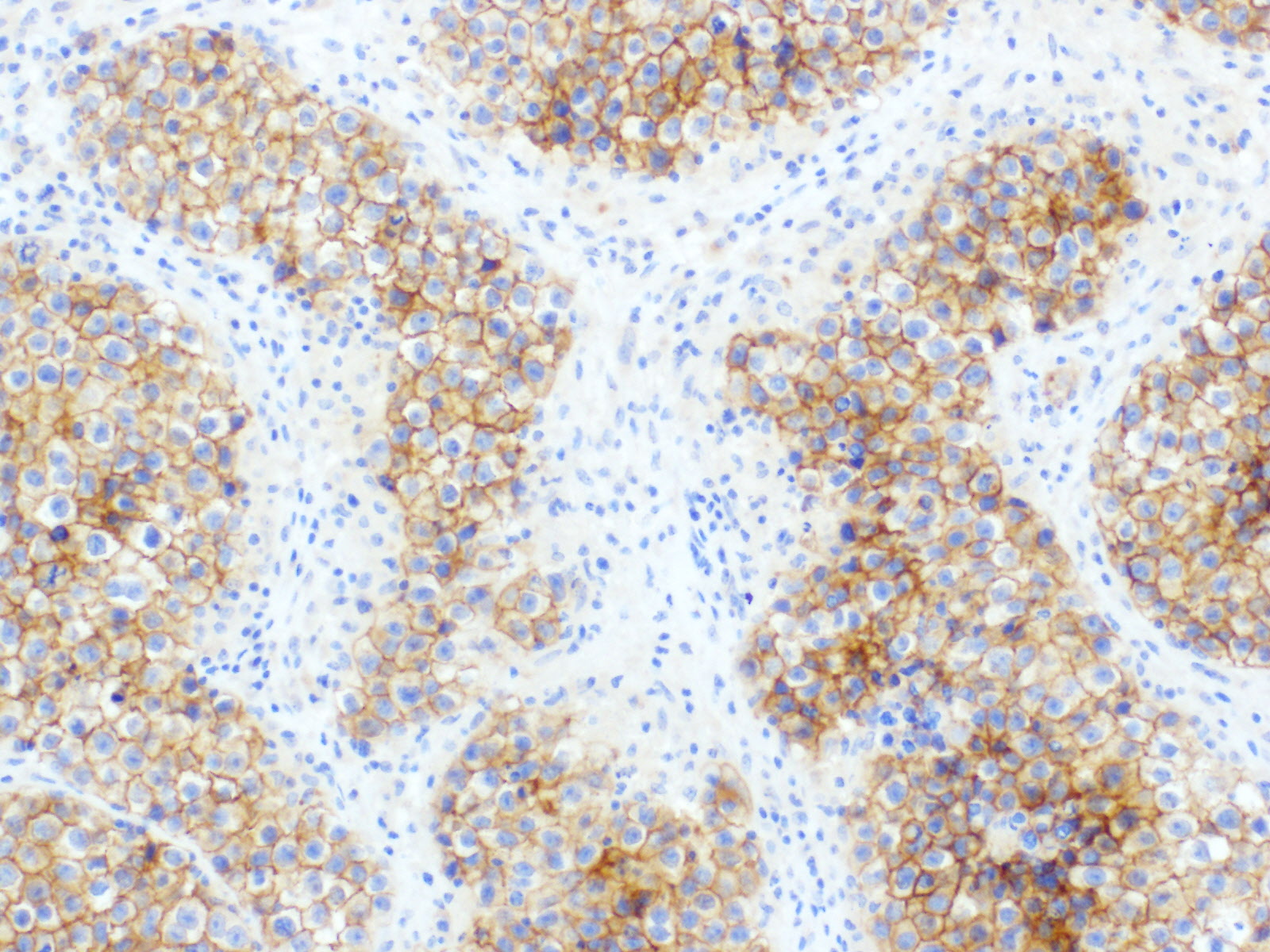

Supplement: Supplementary file 1 [file Presentation1.ZIP › 14 composante dysgerminome CD117 pos.JPG]

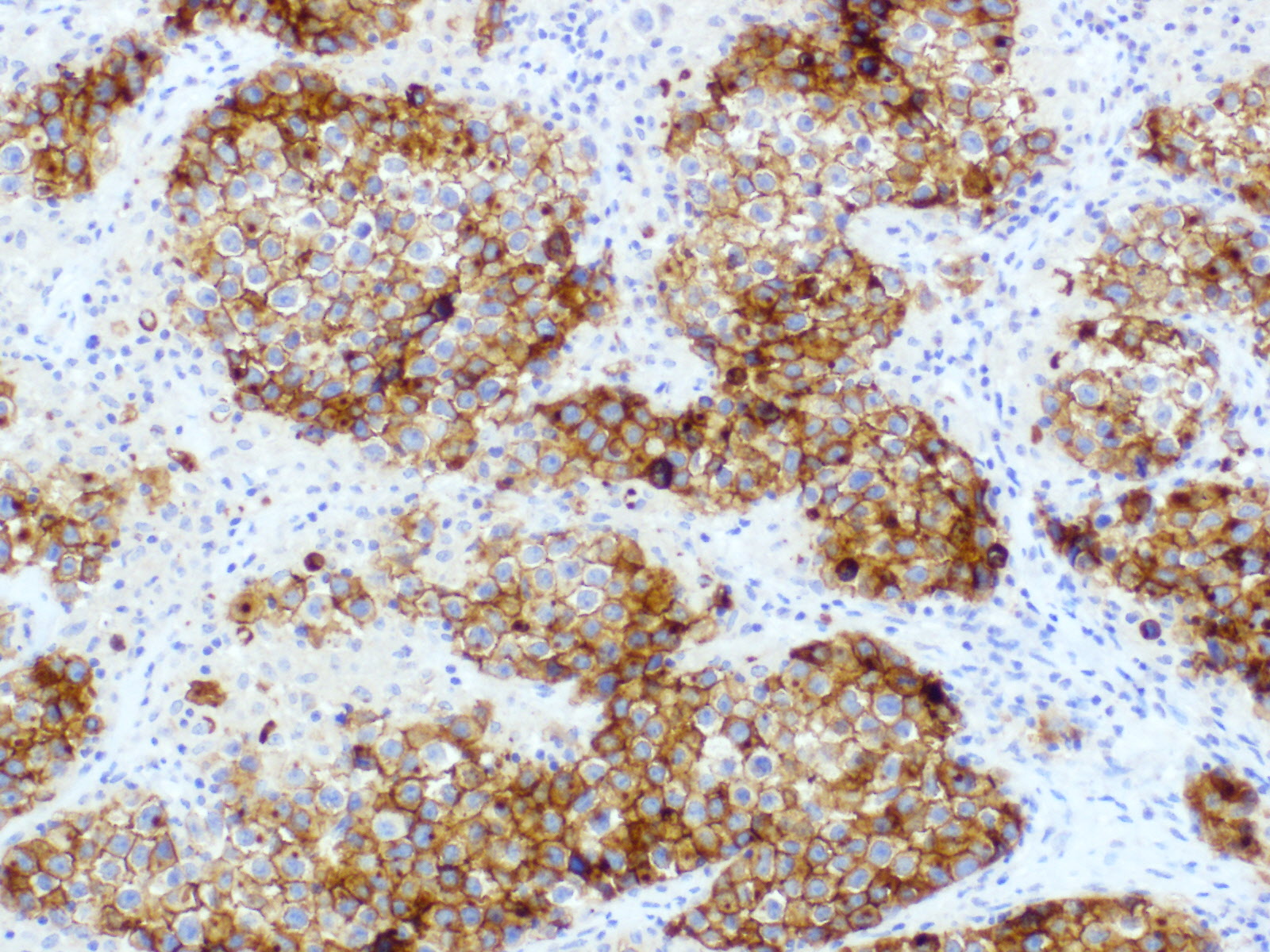

Supplement: Supplementary file 1 [file Presentation1.ZIP › 15 composante dysgerminome PLAP pos.JPG]

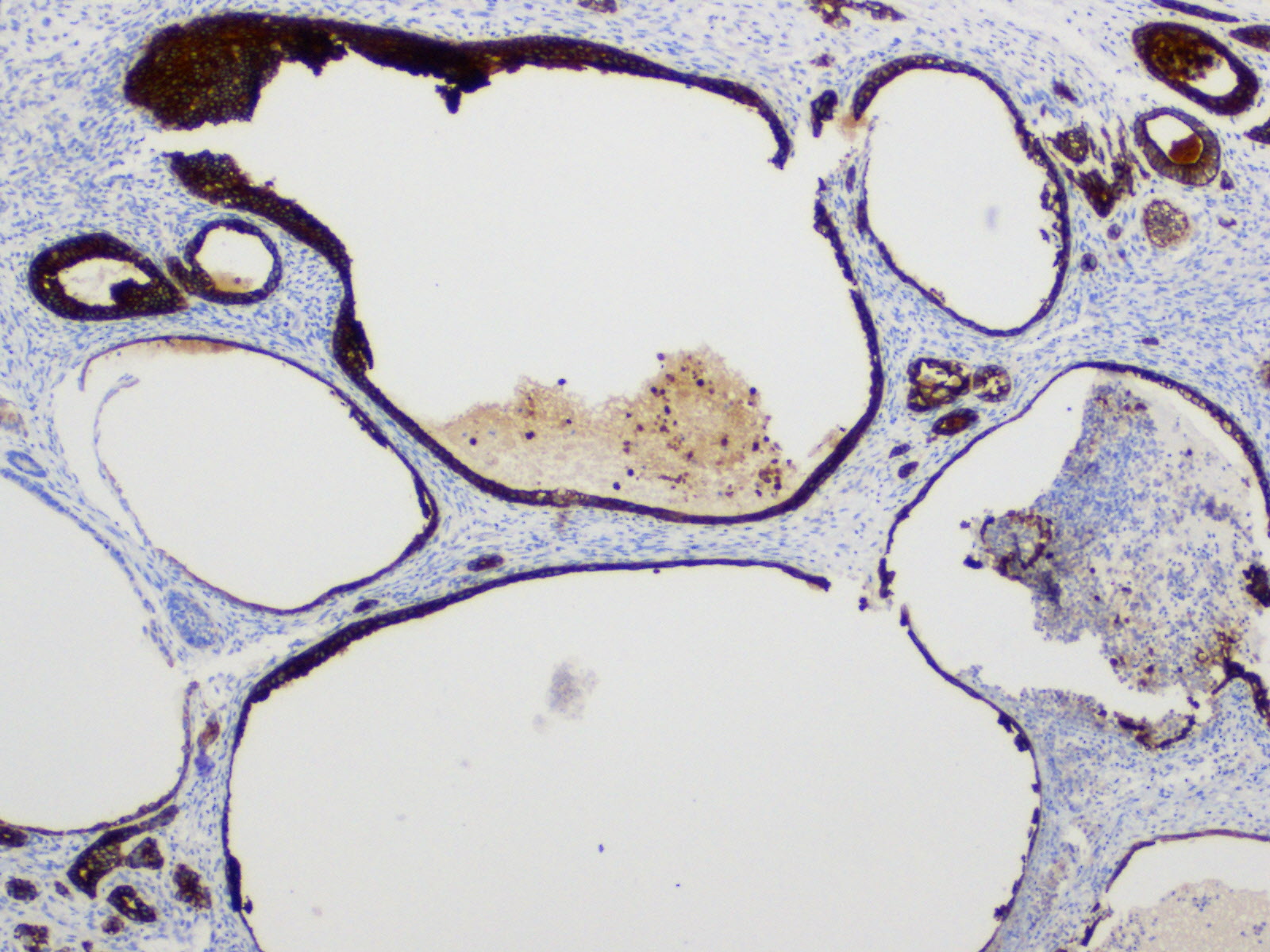

Supplement: Supplementary file 1 [file Presentation1.ZIP › 16 composante tératome imm kératines pos.JPG]

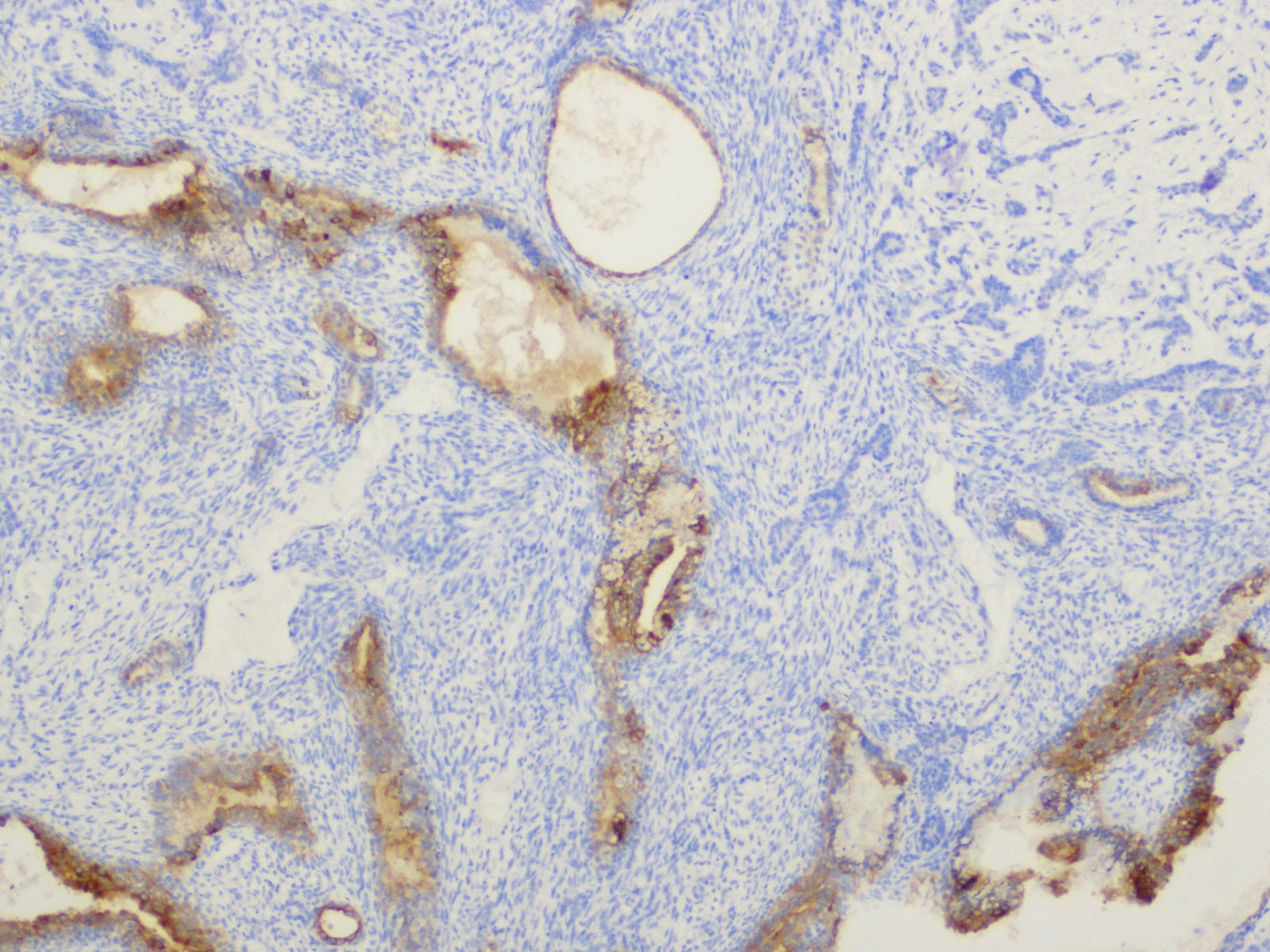

Supplement: Supplementary file 1 [file Presentation1.ZIP › 17 tératome imm EMA pos, tum vittelline EMA négatif.JPG]

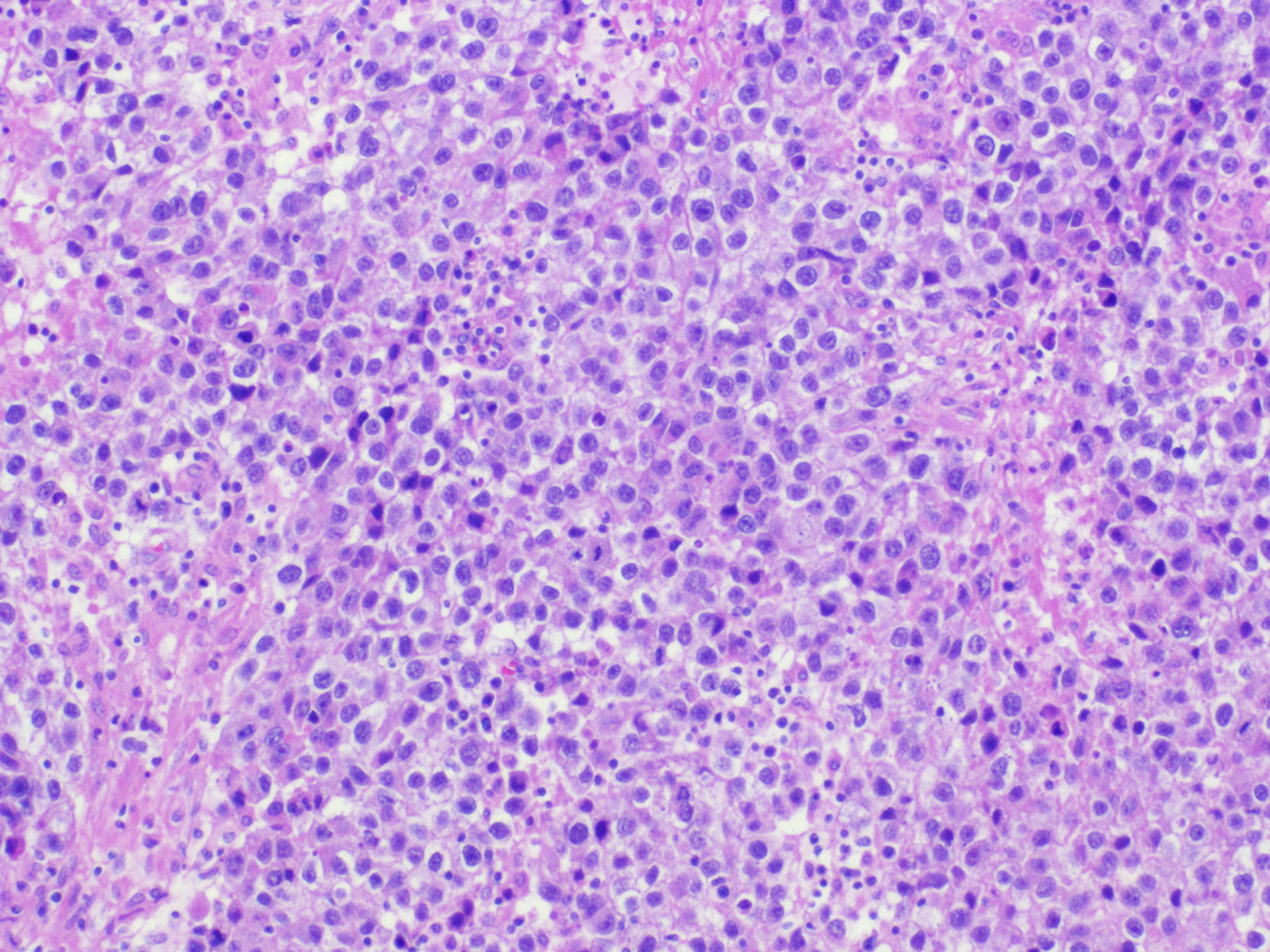

Supplement: Supplementary file 1 [file Presentation1.ZIP › 2 composante dysgerminome.JPG]

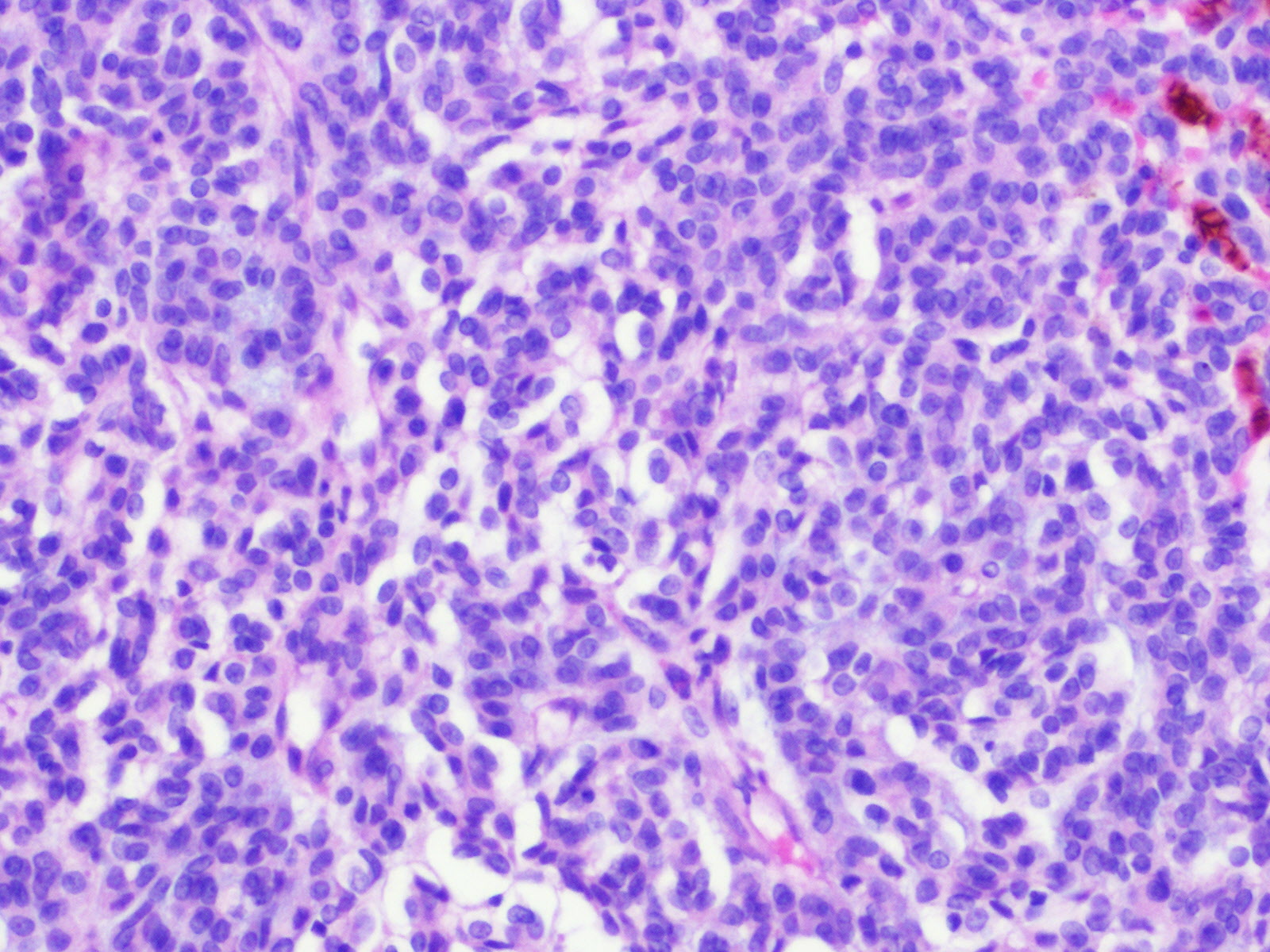

Supplement: Supplementary file 1 [file Presentation1.ZIP › 2 tum granulosa HE x20.JPG]

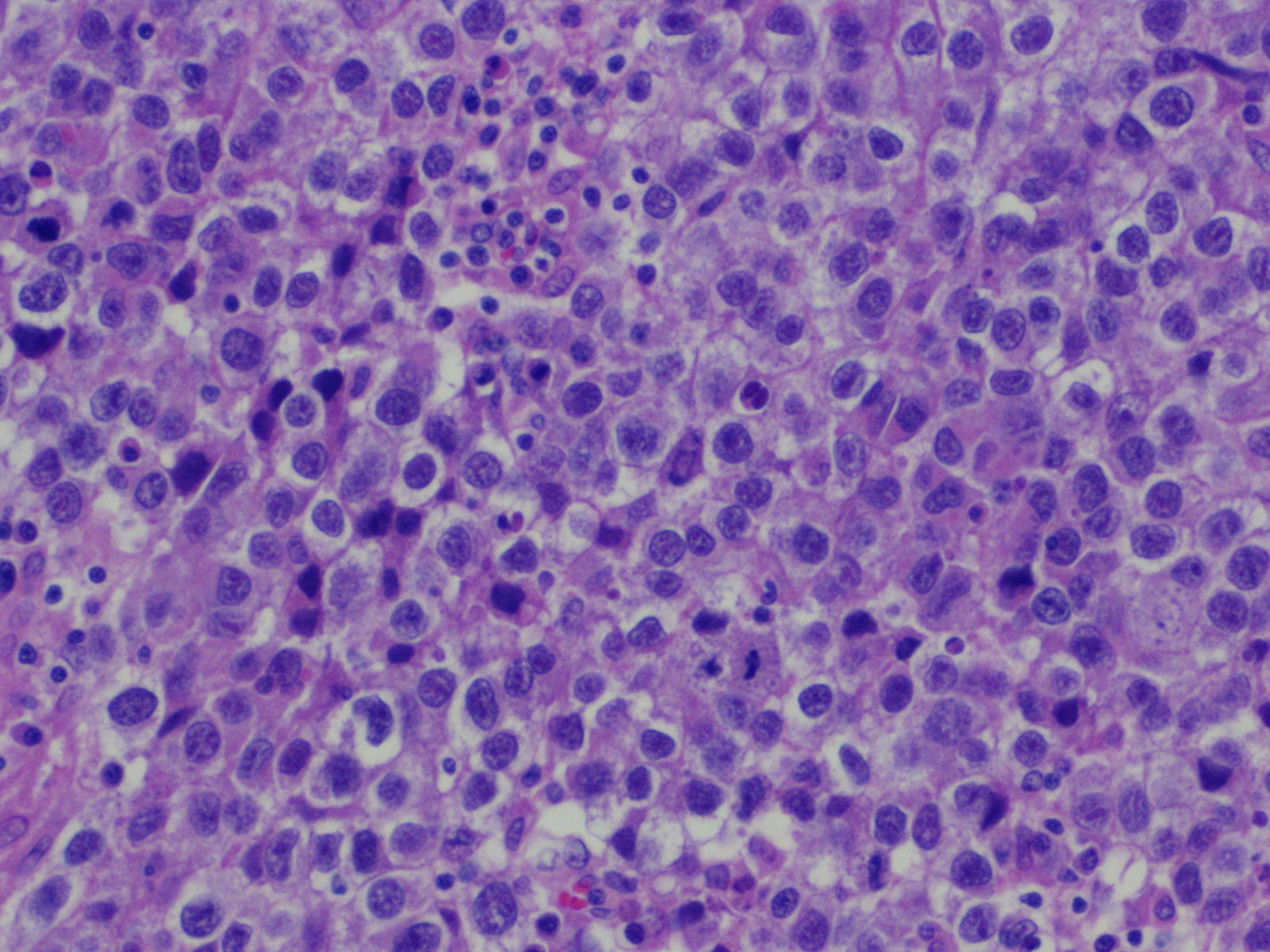

Supplement: Supplementary file 1 [file Presentation1.ZIP › 3 composante dysgerminome.JPG]

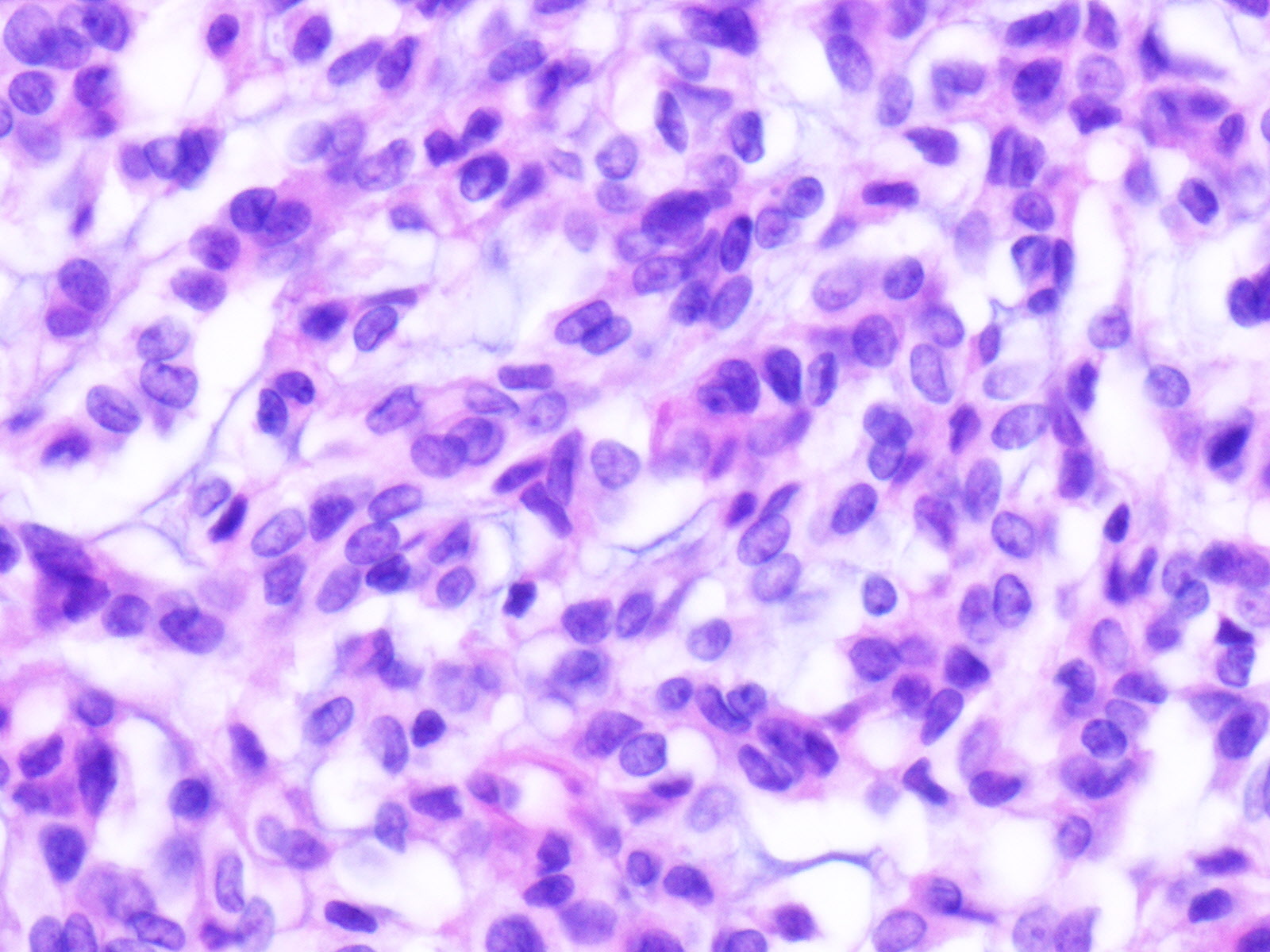

Supplement: Supplementary file 1 [file Presentation1.ZIP › 3 tum granulosa HE x40.JPG]

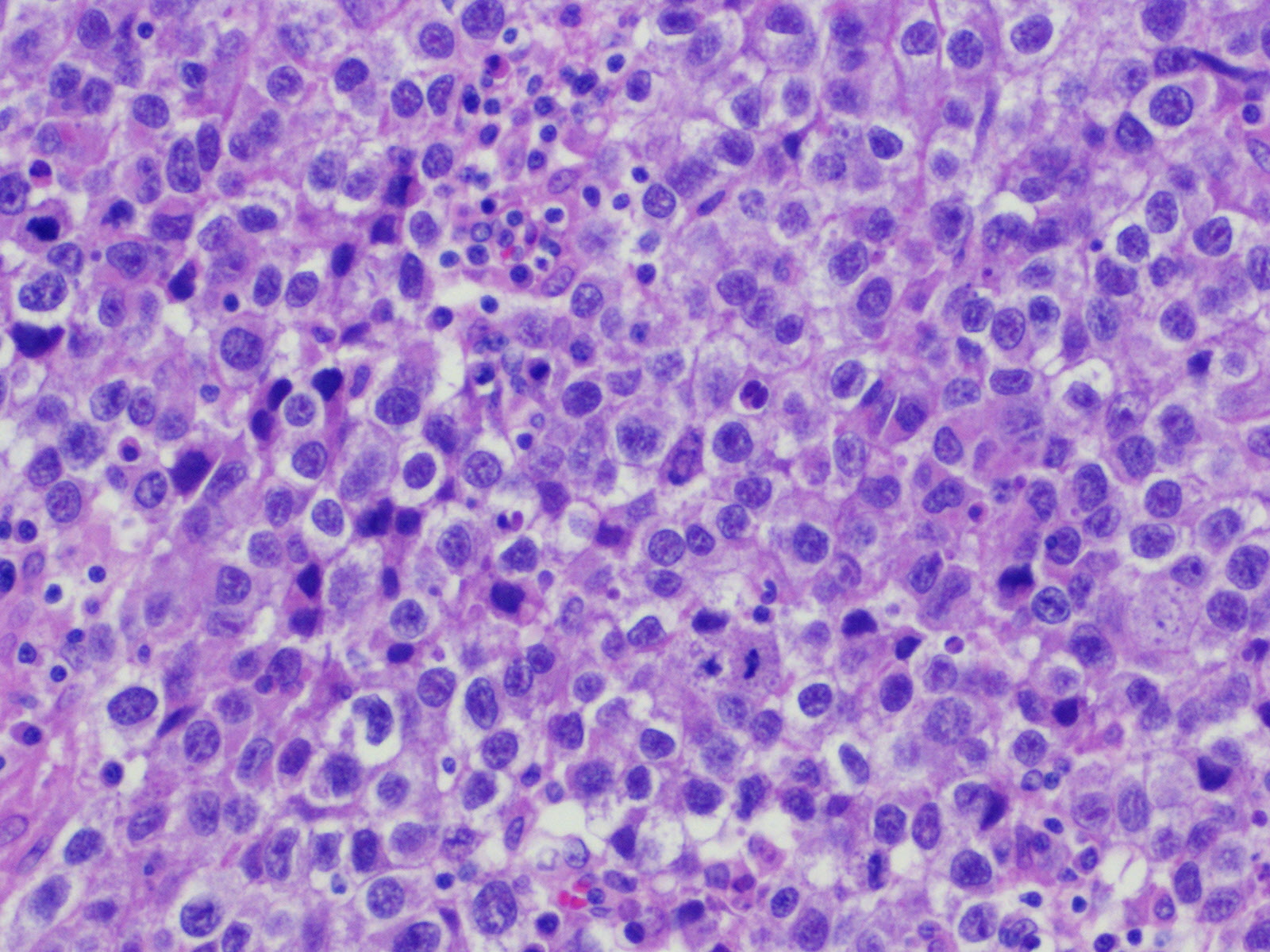

Supplement: Supplementary file 1 [file Presentation1.ZIP › 4 composante dysgerminome.JPG]

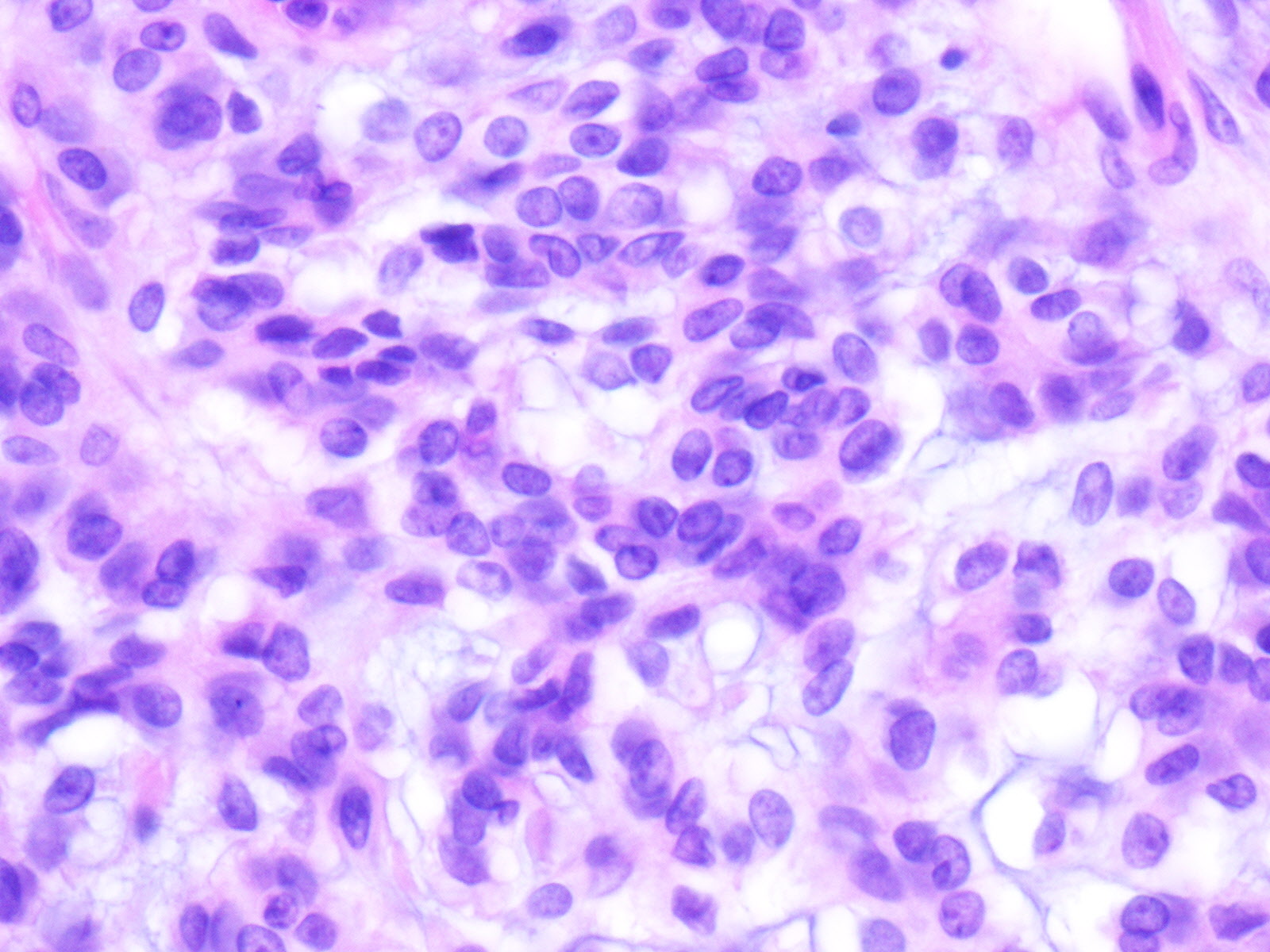

Supplement: Supplementary file 1 [file Presentation1.ZIP › 4 Tum granulosa HE x40.JPG]

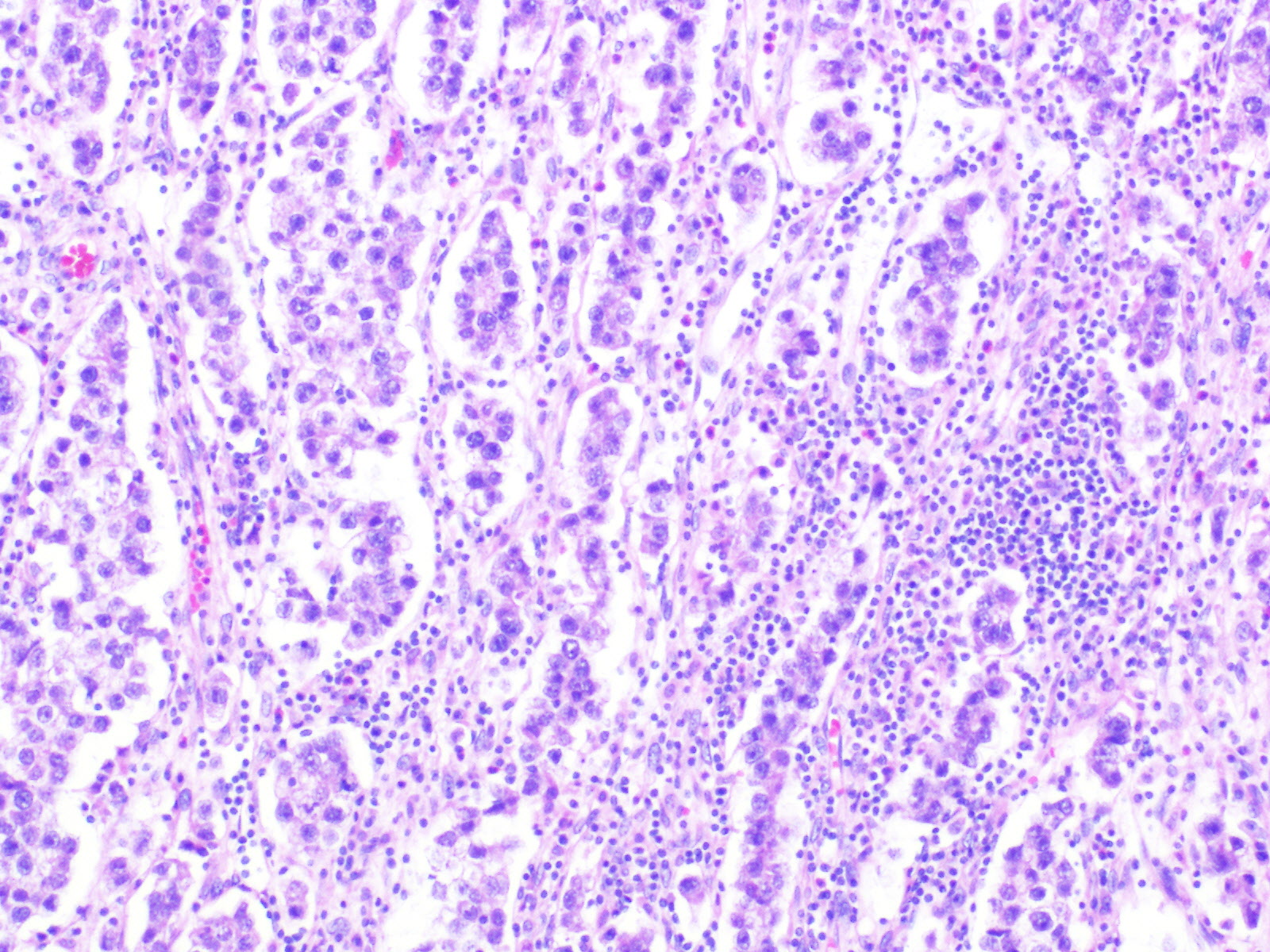

Supplement: Supplementary file 1 [file Presentation1.ZIP › 5 composante dysgerminome.JPG]

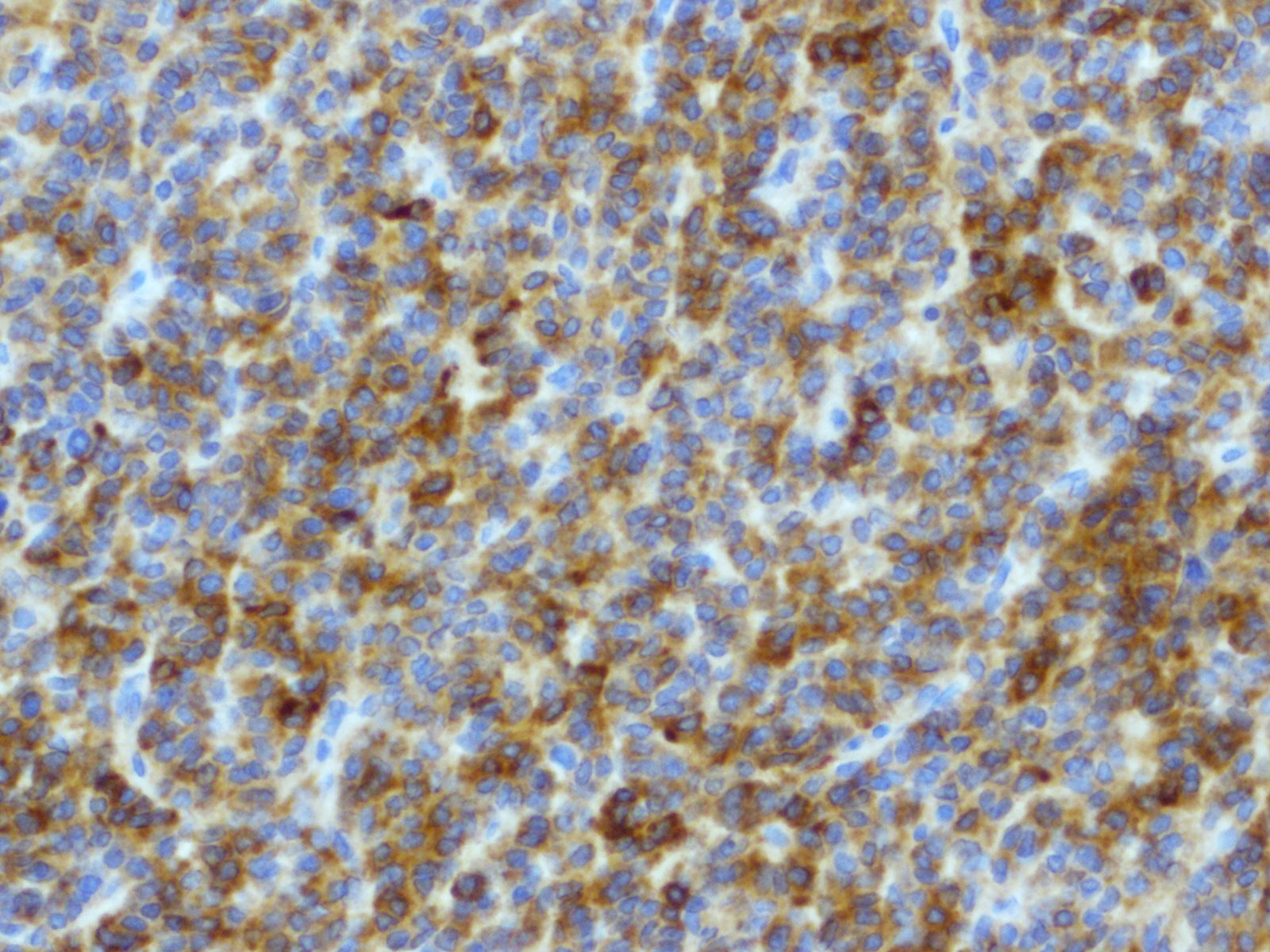

Supplement: Supplementary file 1 [file Presentation1.ZIP › 5 Tum granulosa Inhibine pos.JPG]

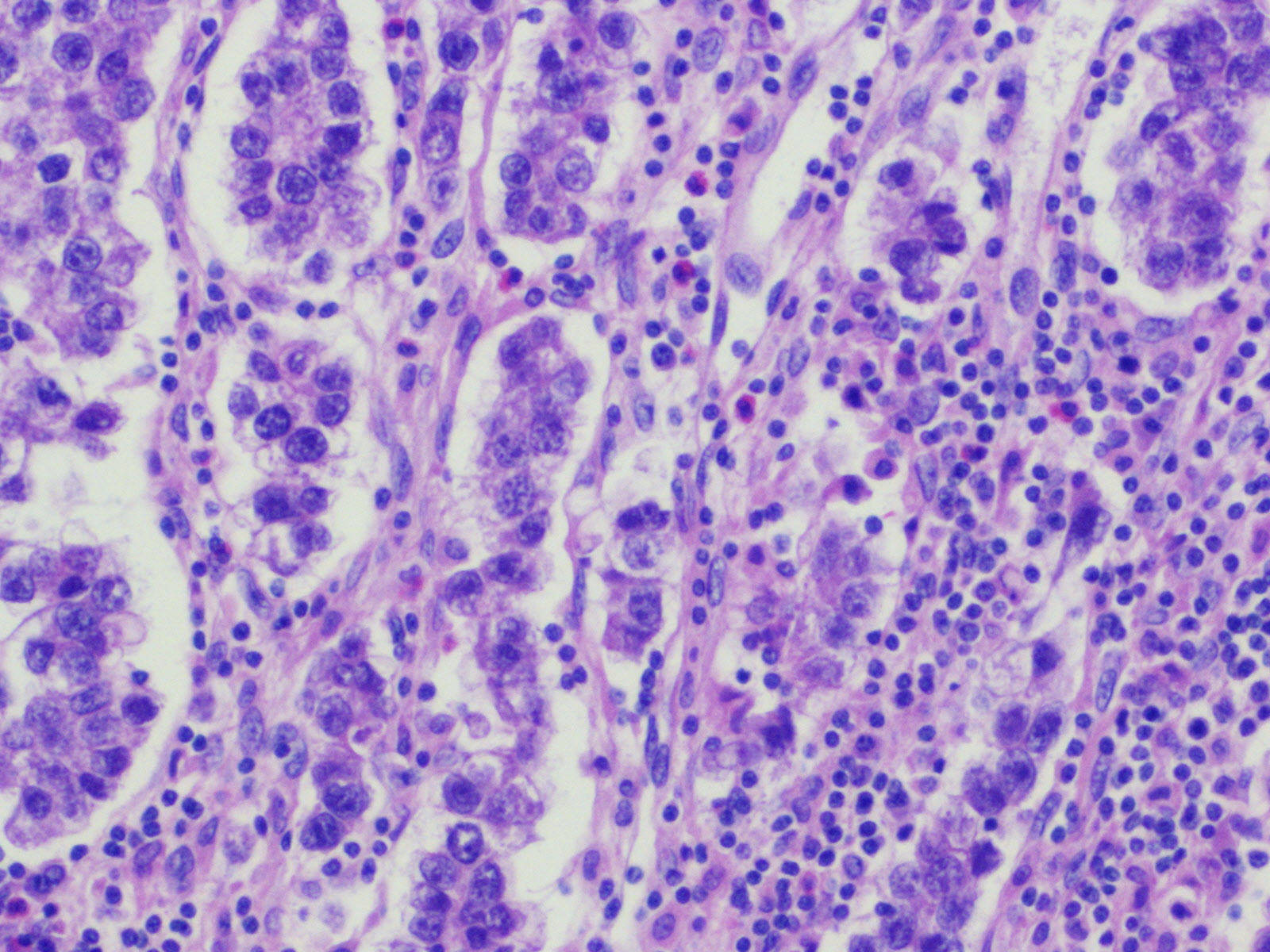

Supplement: Supplementary file 1 [file Presentation1.ZIP › 6 composante dysgerminome.JPG]

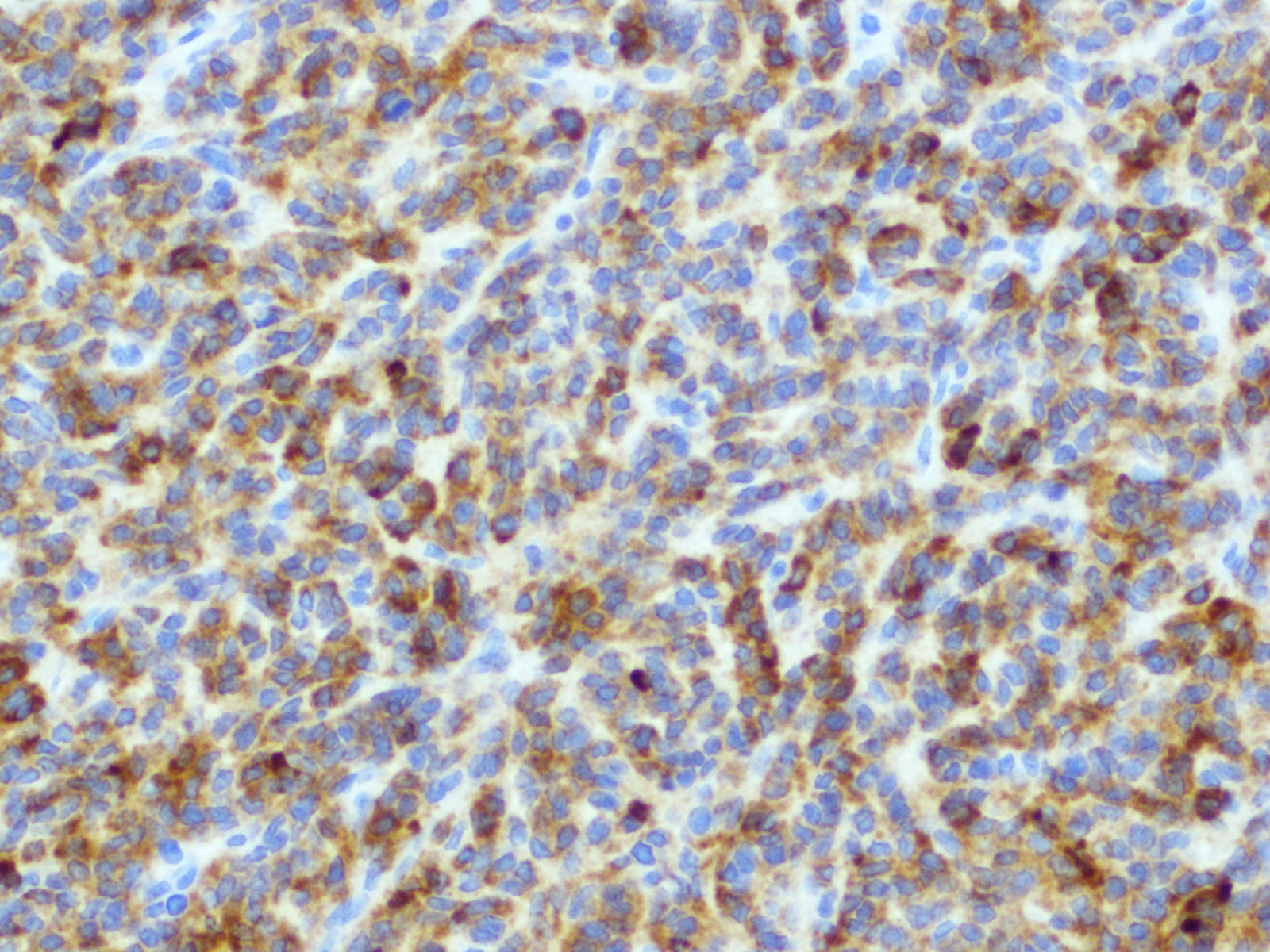

Supplement: Supplementary file 1 [file Presentation1.ZIP › 6 Tum granulosa Inhibine pos.JPG]

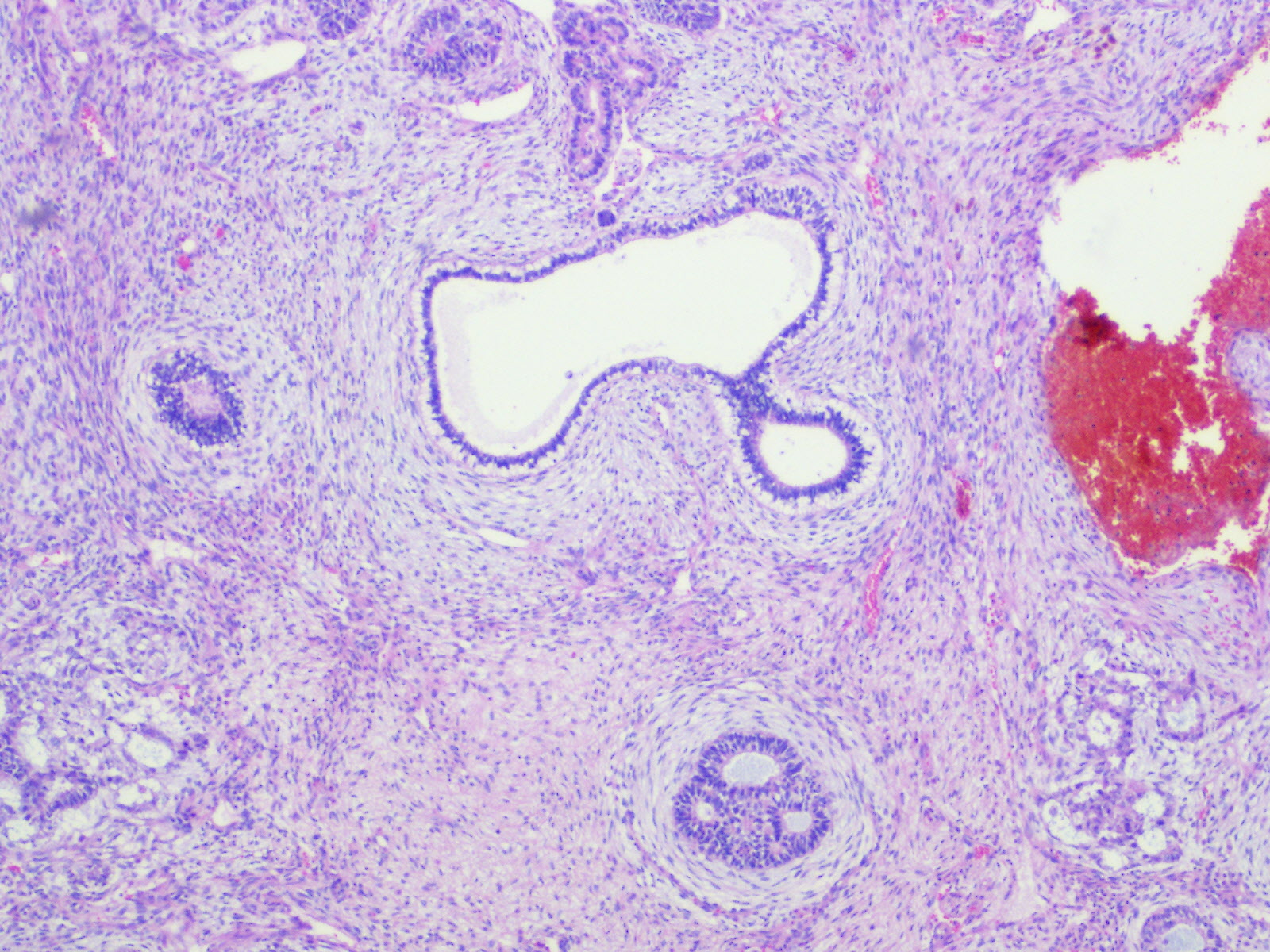

Supplement: Supplementary file 1 [file Presentation1.ZIP › 7 composante tératome immature.JPG]

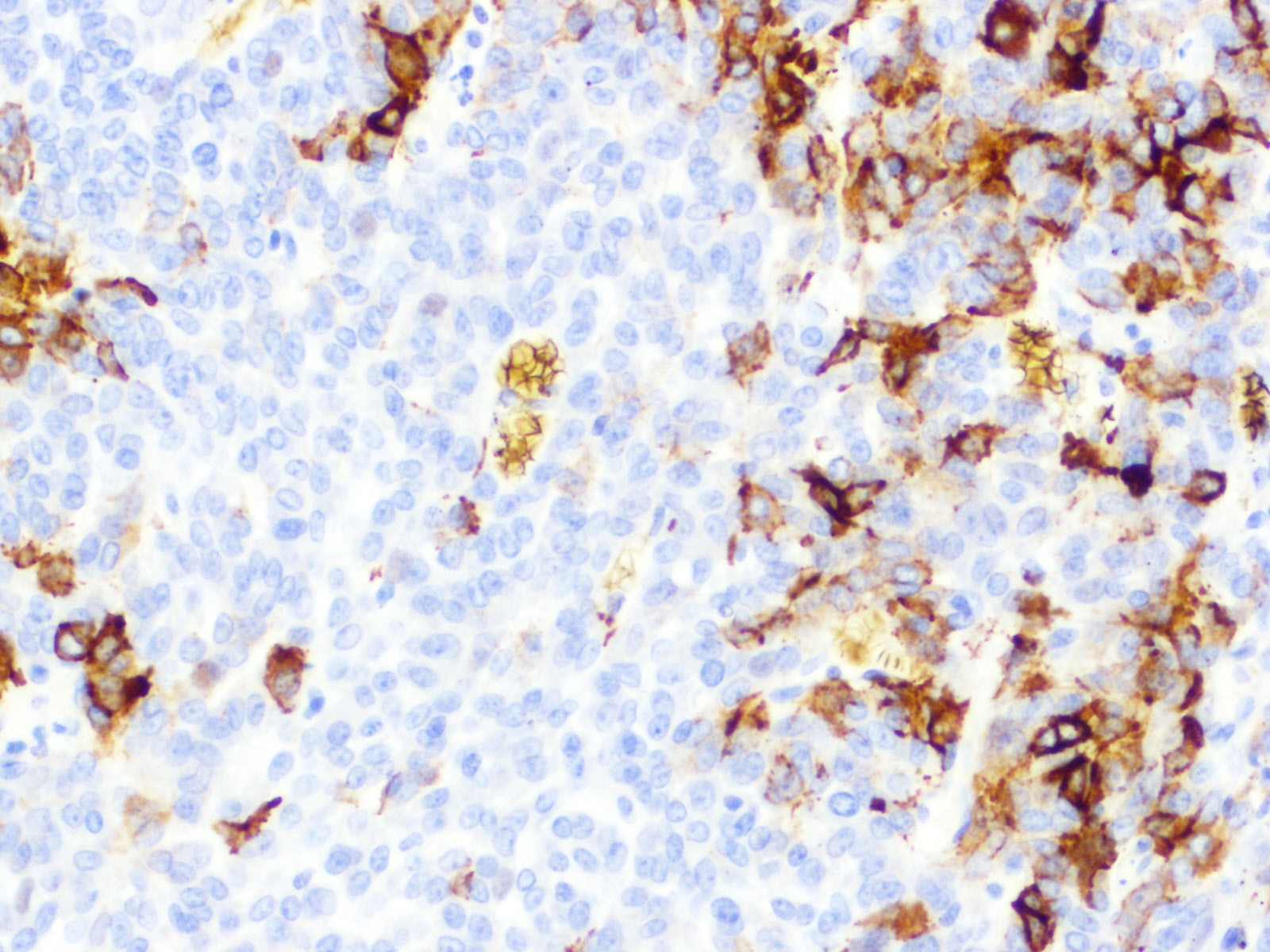

Supplement: Supplementary file 1 [file Presentation1.ZIP › 7 Tum granulosa actine lisse focal pos.JPG]

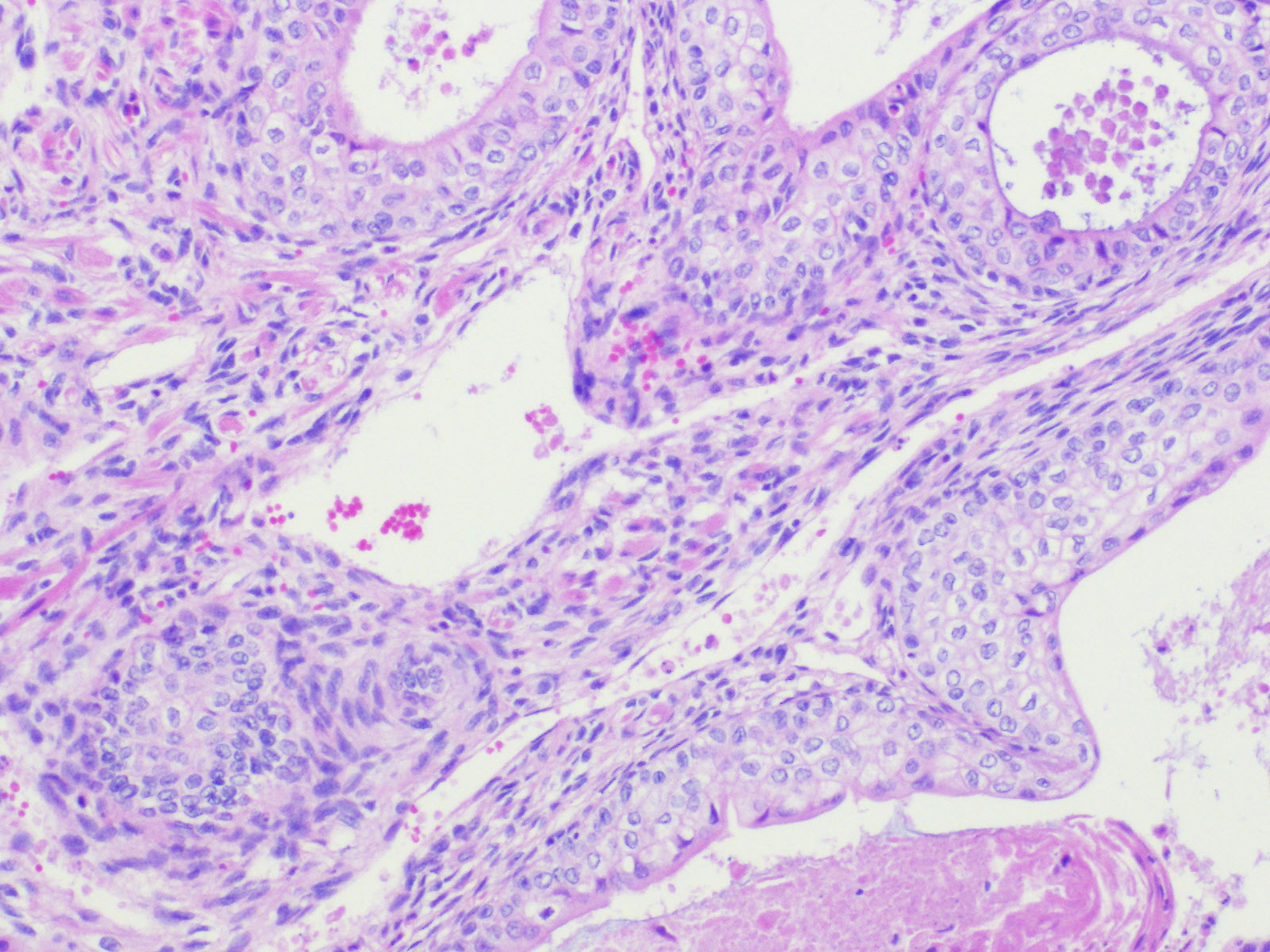

Supplement: Supplementary file 1 [file Presentation1.ZIP › 8 composante tératome immature.JPG]

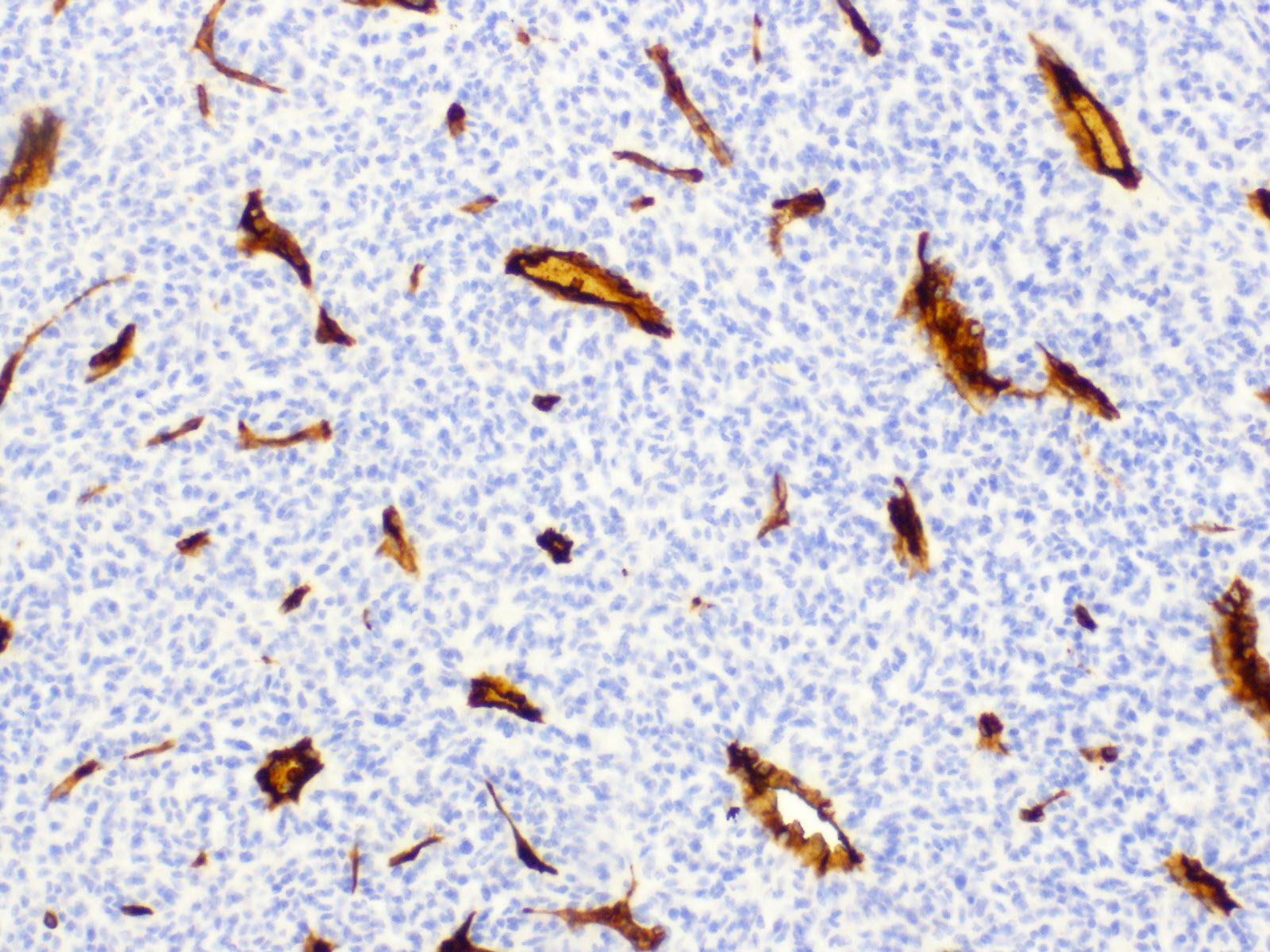

Supplement: Supplementary file 1 [file Presentation1.ZIP › 8 tum granulosa CD34 nég.JPG]

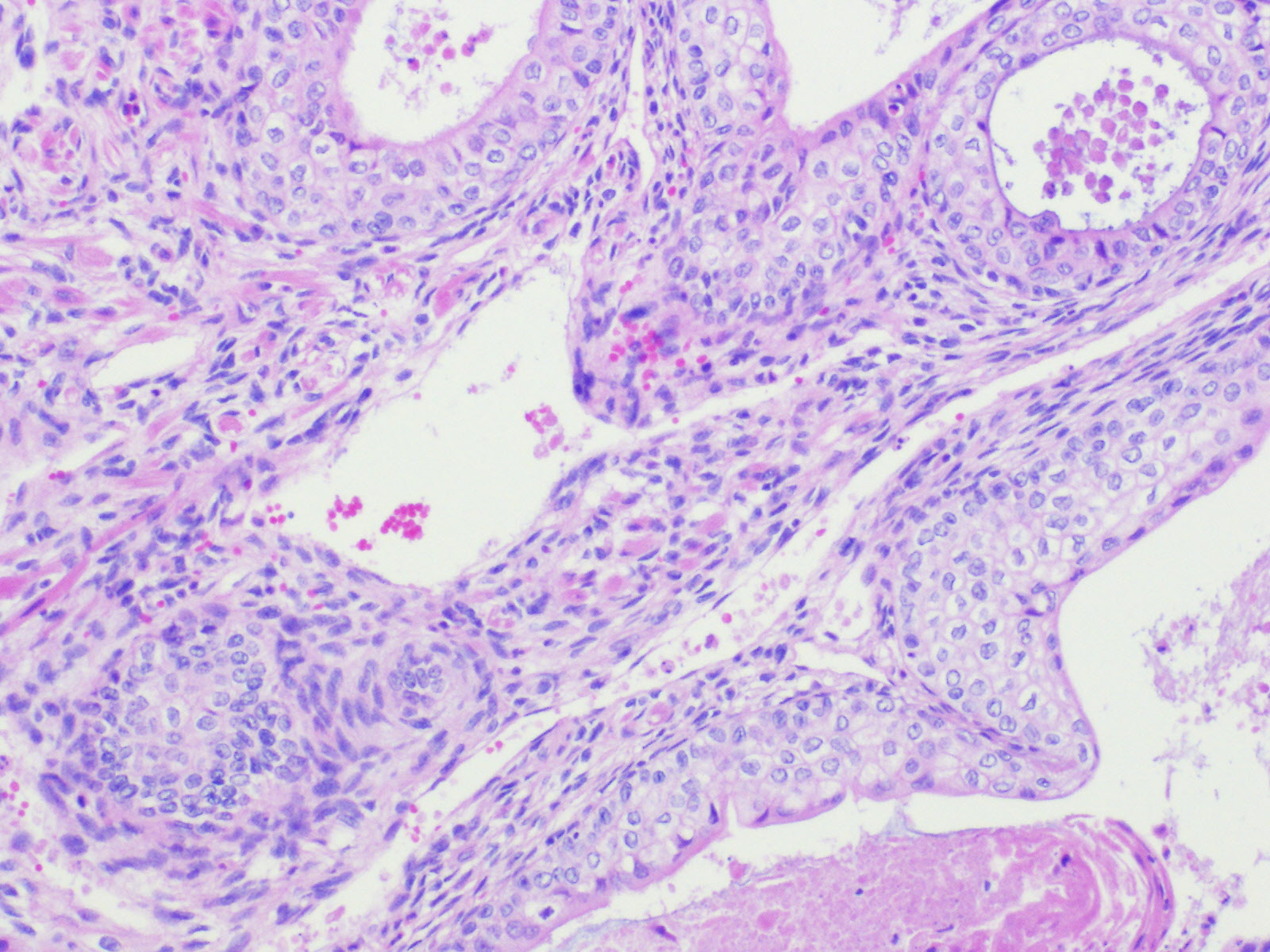

Supplement: Supplementary file 1 [file Presentation1.ZIP › 9 composante tératome immature.JPG]

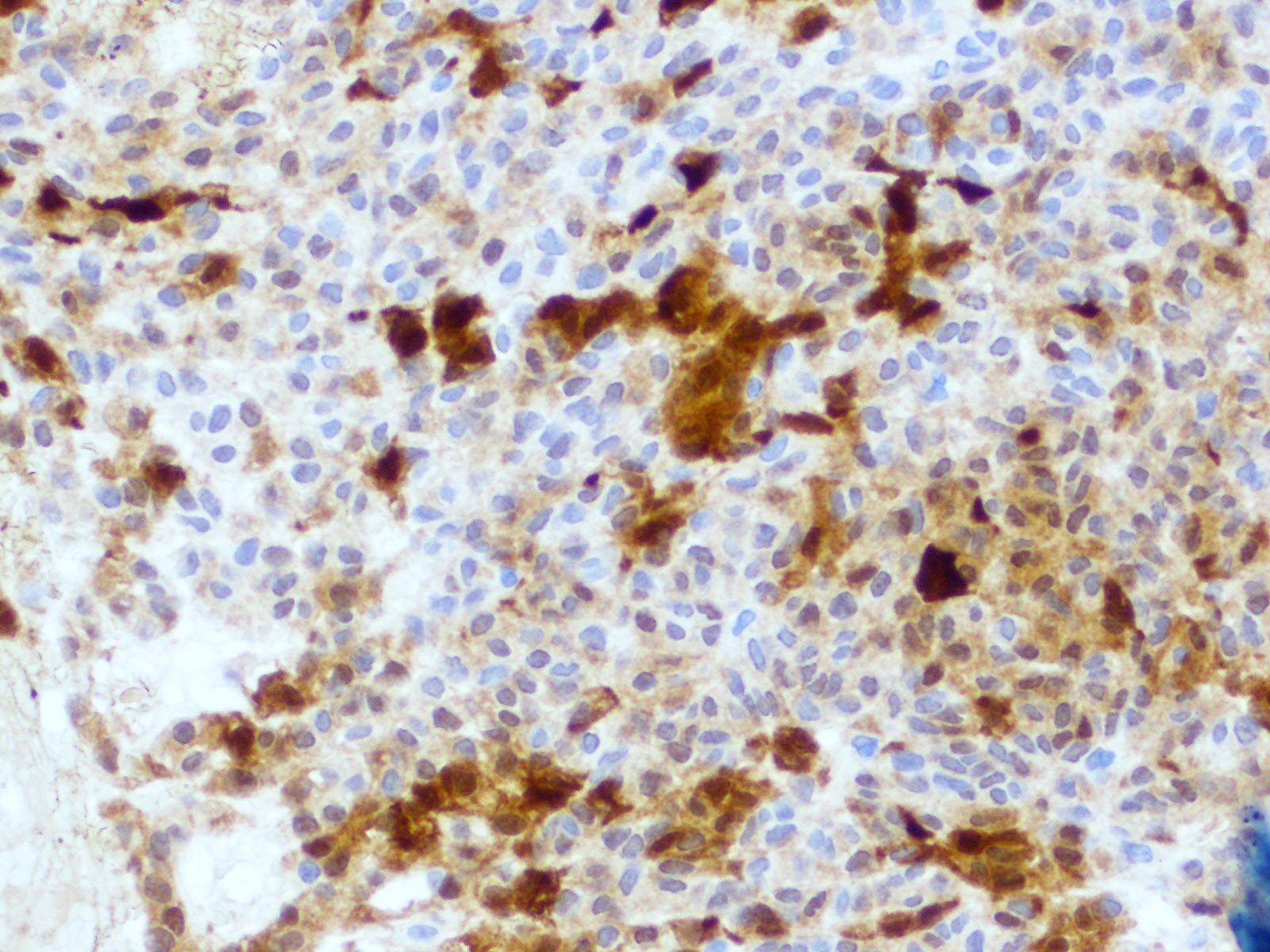

Supplement: Supplementary file 1 [file Presentation1.ZIP › 9 Tum granulosa calrétinine pos.JPG]
